# Role of microbiota in pain: From bench to bedside

**DOI:** 10.1002/imo2.58

**Published:** 2025-02-11

**Authors:** Huiguang Ren, Bo Cao, Qixuan Xu, Ruiyang Zhao, Hanghang Li, Bo Wei

**Affiliations:** ^1^ Department of General Surgery, First Medical Center Chinese PLA General Hospital Beijing China; ^2^ Medical School of Chinese PLA Beijing China

**Keywords:** fecal microbiota transplantation, microbiota, neural circuit, pain, probiotics

## Abstract

Interactions between the microbiota and host have been proven to be critical regulators of homeostasis, and pain perception is no exception. Emerging evidence has identified the mechanisms by which microbiota dysbiosis contributes to hyperalgesia and revealed the potential value of microbiota‐associated therapies in pain management. Herein, the authors introduce the basic knowledge of pain and microbiota for readers who are not simultaneously majoring in these two fields. The clarified mechanisms underlying the regulation of pain by the microbiota are outlined in terms of three ways. This review summarizes the current advancements in pain management and microbiology research for clinicians who wish to focus on this area. Probiotics, fecal microbiota transplantation, and other methods of microbiota modulation for pain management have entered clinical translation. The authors further propose the present limitations and prospects for high‐quality development of preclinical and clinical investigations. Importantly, despite the large amount of attention given to gut bacteria, this review also puts forward great expectations on the role of nongut and nonbacterial microbiota in pain sensation. Efforts to decipher the mechanisms of microbiota functions will help to promote achievements in pain management from bench to bedside.

## Highlights

- Pain, a major clinical issue, is significantly associated with microbiota, highlighting their crucial role in regulating host biology and pathology.
- The mechanisms by which microbiota modulates pain perception can be divided into three aspects, including functions of microbiota‐derived metabolites, immunoregulation, and direct stimulation on neurons.
- Emerging therapeutic strategies for microbiota intervention, such as probiotics, fecal microbiota transplantation, and diet adjustment, have exhibited great potential in pain management.
- The rapid advancement in interdisciplinary and multi‐omics technologies accelerates pain research and facilitates the clinical application of microbiota in pain diagnosis and treatment.

## Graphical abstract

Pain is closely linked to microbes. The microbiota modulates pain perception through three mechanisms: microbiota‐derived metabolites, immunoregulation, and direct stimulation on neurons. Therapeutic strategies like probiotics, fecal microbiota transplantation, and dietary changes show significant potential in pain management. Interdisciplinary advancements are accelerating research and clinical applications in this field.

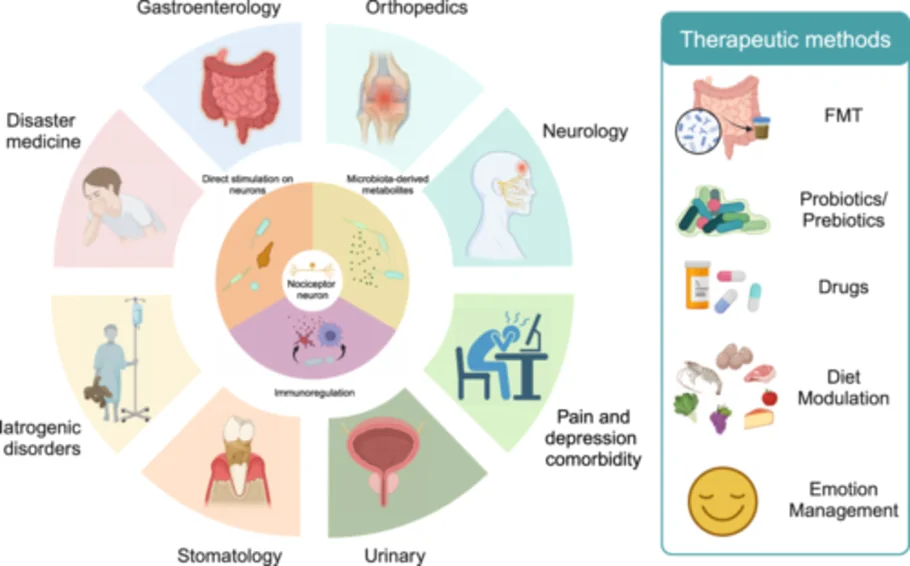

## 1 INTRODUCTION

Pain is a subjective and distressing sensation. Chronic pain impairs patients' work ability to varying degrees, depletes substantial medical resources, and results in imposing a considerable socioeconomic burden. Additionally, chronic pain significantly affects psychological well‐being, leading to issues such as depression, anxiety, and social withdrawal [1].

The human microbiome consists of approximately 4 × 10^13^ microbes [2]. Thousands of bacterial strains, together with archaea, viruses, fungi, protozoa, and helminths, collectively form the intricate ecosystem. Bacteria, one of the important components of the microbiome, have gene loads that are two to three orders of magnitude larger than those of human genes [3], accounting for some potential areas in future studies.

Microorganisms play a critical role in modulating both physiological and pathological processes in eukaryotes, and the mechanisms by which they influence pain perception are increasingly being elucidated. New therapies against gut microbiota dysregulation have presented great value in pain management. Other than flourishing research on gut microbiota, extensive investigations are being conducted on the functions of non‐gut commensal microbiota, which represents a crucial avenue for future advancements in pain diagnosis, evaluation, and treatment.

This review will briefly introduce the basic knowledge concerning pain research. Subsequently, we concentrate on the mechanisms by which microorganisms are involved in pain, as well as the clinical implications of investigating their role in pain. The study limitations and future perspectives will be proposed for the better development of microbiota‐related diagnosis and therapies for pain management.

## 2 BASIC CIRCUITS OF PAIN PERCEPTION

Pain is a multifaceted phenomenon encompassing both physiological and psychological aspects, triggered by noxious stimuli and involving the body's intricate pain mechanisms. Categorically, pain can be classified into nociceptive, neuropathic, and nociplastic types based on its pathogenesis. Nociceptive pain primarily arises from tissue damage and is commonly observed in conditions like primary osteoarthritis. Neuropathic pain manifests as sensory abnormalities resulting from nerve damage, exemplified by neuralgia associated with herpes zoster infection. Nociplastic pain emerges due to altered nervous system sensitivity and abnormal processing of pain signals, as seen in abdominal discomfort experienced in irritable bowel syndrome (IBS) [1].

According to physiological characteristics, the process of pain generation can be delineated into four principal phases: transduction, transmission, modulation, and perception. The cell bodies of nociceptive neurons are located in the dorsal root ganglia (DRG) and trigeminal ganglia. Nociceptors refer to the free nerve endings of the axons of these neurons that innervate the skin, mucous membranes, and vascular connective tissue. Nociceptors possess key signal transduction molecules, such as G‐protein‐coupled receptors (GPCRs), transient receptor potential (TRP) channels, Nav channels. They have the ability to respond to chemical, thermal, and mechanical stimuli by generating a transmembrane ionic current known as the receptor potential. Upon reaching the axonal terminal, sufficient depolarization triggers action potentials that propagate along the nerve fibers, known as the transduction process. Pain signals are transmitted through Aδ and C fiber afferents. Aδ fibers, which are myelinated somatic afferents, facilitate the rapid transmission of sharp and well‐localized pain. Conversely, C fibers, being unmyelinated somatic afferents with a higher activation threshold, conduct slow, dull, and poorly localized pain sensations [4]. Certain signaling molecules can decrease the threshold for action potential initiation, leading to hyperalgesia, which is the pain modulation process [5]. Following synaptic exchange, electrical signals primarily ascend to the thalamus via the spinothalamic and trigeminal pathways before being projected to the somatosensory cortex [4]. Ultimately, pain perception occurs in the cortex.

## 3 MILESTONES IN PAIN RESEARCH ON MICROBIOTA

**Figure 1:**
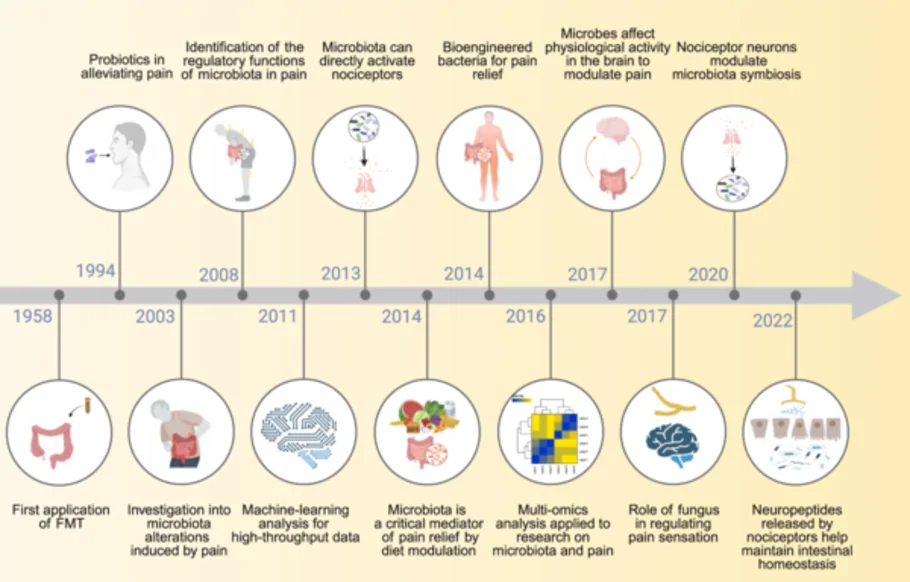
The brief timeline of milestones in the research on the roles of microbiota in pain. In 1958, FMT was first applied in clinical practice, initiating a new era of the research on microbiota functions in pain. The investigations into probiotics efficacies in pain manipulation further promoted the development of related studies. In the new century, the research has stepped into the rapid‐progress stage. The bilateral interactions between microbiota and neurons have been proved to contribute to pain modulation. Modern technologies, like artificial intelligence, bioengineering and multi‐omics analysis, have accelerated the research progress. The remarkable advances further spawned novel therapeutic regimens for pain relief. The historical milestones are highlighted. FMT, fecal microbiota transplantation.

There has been a long history of the research on the relationships between microbiota and pain (Figure 1). Over a thousand years ago, Chinese doctors found that the administration of Huanglong decoction, which is essentially the juice of feces collected from healthy persons, can effectively improve gut dysfunctions. The naïve understanding of the gut microbiota functions in abdominal pain laid the elementary foundations for the modern research [6]. In 1958, Eiseman et al. first successfully treated patients with pseudomembranous enterocolitis using fecal enema [7]. This momentous event opened a new chapter for the investigations into regulatory functions of microbiota. Subsequently, this therapeutic approach was officially named as fecal microbiota transplantation (FMT). The progress of research in this field entered a slow‐paced stage over the next few decades, partly due to the relatively underdeveloped biotechnologies and limited understanding of microbiota functions. In 1994, probiotic therapy began to be used in pain manipulation [8]. The optimal effectiveness and safety greatly boosted the research enthusiasm on the development and clinical translation of probiotics.

The research has accelerated into the fast lane in the 21st century. The microbiota dysbiosis in patients with IBS was precisely depicted using culture biochemical methods and mass fragmentography in 2003 [9], which first established the correlations between specific microbial strains and pain. After 5 years, Amaral et al. found that inflammatory hypernociception was significantly alleviated in the absence of the microbiota, which was further reversed by microbiota recolonization [10]. This pioneering study first provided direct evidence on the regulatory functions of microbiota in pain. In 2011, one study first employed artificial intelligence techniques to analyze high‐throughput microbiome data. The gut microbiome signatures were able to accurately classify the subtypes of IBS patients. The abundances of some bacterial strains were found to be correlated with pain intensities [11].

Important advances have also been made in the study of pain‐modulating mechanisms. Before the study conducted by Chiu et al. in 2013, it was widely believed that microbiota activated nociceptors only through inducing inflammatory responses or secreting specific metabolites. However, their study showed that gut microbiota could directly stimulate nociceptor neurons and produce pain sensations [12]. The blockbuster conclusion changed such stereotype of researchers. One year later, two landmark studies came on the scene. The first study demonstrated the pivotal role of microbiota in pain modulation through dietary manipulation [13], providing robust support for further investigations into the analgesic effects of diet. In addition, a significant milestone was achieved with the generation and initial application of bioengineered bacteria possessing enhanced analgesic properties for pain relief. This seminal attempt provided a novel direction in microbial therapies against pain [14]. Combinational analysis of microbiome and metabolomics was first conducted in 2016, which started the multi‐omics era of the research on microbiota and pain [15]. In 2017, Luczynski et al. first proved that microbiota regulated pain sensation through changing brain structures and functions. This study further broadened our horizons on the regulatory functions of microbiota [16]. Moreover, all the previous studies at that time focused on the functions of bacteria. The potential roles of non‐bacterial microbiota were neglected. In the same year, Botschuijver et al. indicated that fungal dysbiosis also contributed to the development of pain [17]. A significant milestone was the identification of the regulatory roles of nociceptors on microbiota in 2020. The bilateral interactions between neurons and microbiota were first revealed [18]. In 2022, Zhang et al. and Yang et al. further demonstrated the relationship between microbiota and nociceptors. Microbiota and their products are necessary for the activation of nociceptors, and neuropeptides released by nociceptors help to maintain gut function and microbiota homeostasis and alleviate inflammatory bowel disease (IBD)‐associated pain [19, 20]. The research on microbiota functions in pain has made significant progress, but there are still numerous inquiries regarding the roles of microbiota that need to be addressed. Therefore, further comprehensive investigations should be conducted in this field by leveraging modern biotechnologies.

## 4 MOLECULAR MECHANISMS OF PAIN MODULATION INVOLVING MICROBIOTA

**Figure 2:**
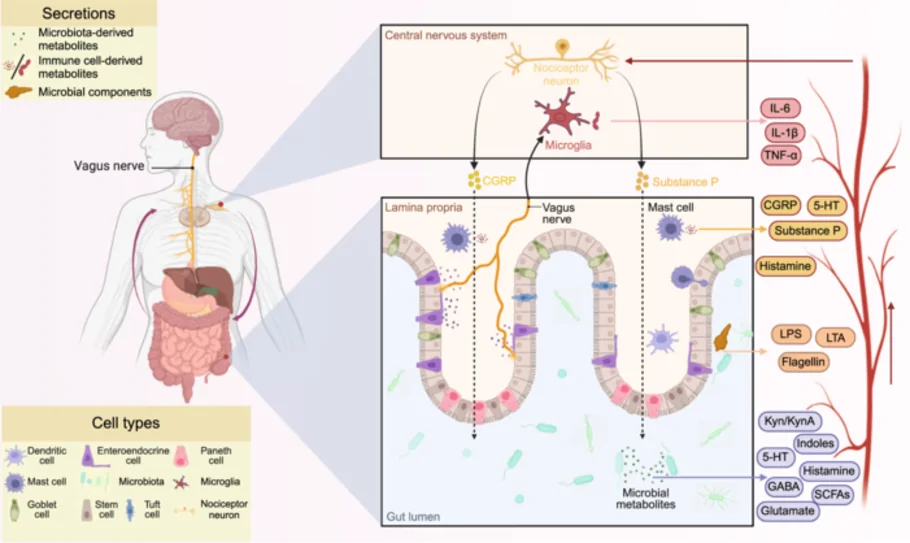
Schematic illustration depicts the stimulation of nociceptor neurons through microbial‐derived metabolites and cytokines produced by activated immune cells via both the circulatory system and vagus nerve. Furthermore, the microbial components can also directly stimulate nociceptor neurons to cause pain. Interestingly, CGRP and SP released by nociceptors neurons play a crucial role in maintaining intestinal flora homeostasis. CGRP, calcitonin gene‐related peptide; Kyn, kynurenine; KynA, kynerunic acid; 5‐HT, serotonin; GABA, γ‐aminobutyric‐acid; SCFAs, short‐chain fatty acids; LPS, lipopolysaccharide; LTA, lipoteichoic acid.

Microbiota‐associated pain modulation involves multiple molecular mechanisms. Metabolites derived from the microbiota stimulate nociceptors to modulate pain as a direct mechanism. The microbiota also activates immune cells to produce cytokines, such as inflammatory factors and chemokines, which is an indirect pain regulatory mechanism. In addition, recent studies have shown that microbiota and specific components directly induce the activation of nociceptors, which transmit harmful stimulus information (Figure 2).

### 4.1 Microbiota‐derived metabolites as direct mechanisms

**Figure 3:**
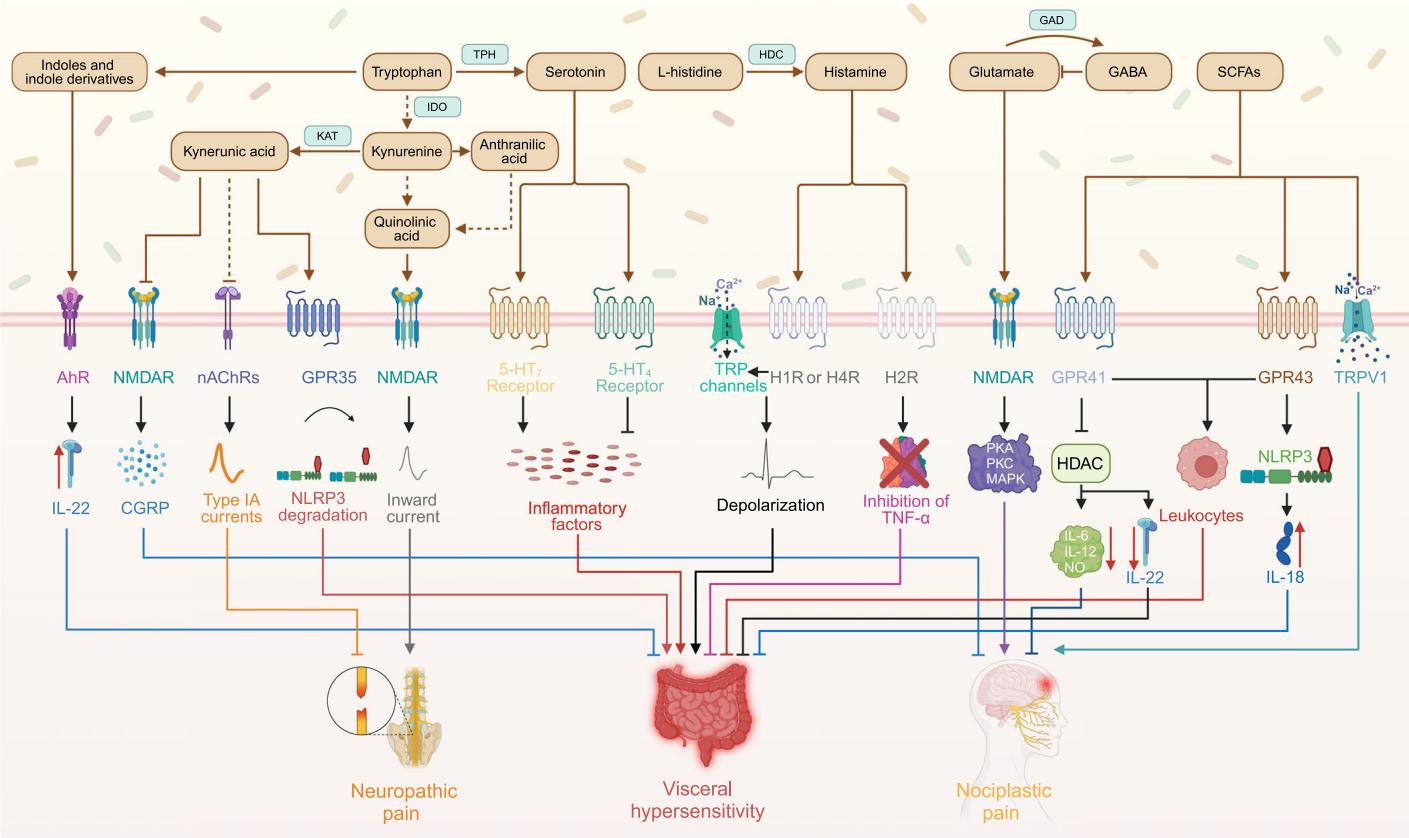
Schematic illustration of the direct mechanisms by which microbiota‐derived metabolites regulate multiple types of pain. The metabolic pathways and receptor interactions for tryptophan, indole, indole derivatives, serotonin, glutamate, GABA, and SCFAs are delineated in a simplified manner. The activation of these receptors triggers a cascade of physiological responses, culminating in the modulation of pain perception. The dashed lines within the tryptophan metabolic pathway signify the omission of intermediate steps, highlighting only the key processes. The dashed arrow connecting KynA and nAChRs suggests a hypothesized yet experimentally unconfirmed pathway for pain modulation. The zigzag line between SCFAs and the TRPV1 illustrates the complexity of their interaction, with both upregulation and downregulation observed in disparate experimental conditions. TPH, tryptophan hydroxylase; 5‐HT, serotonin; IDO, indoleamine 2,3‐dioxygenase; KAT, kynurenine aminotransferase; HDC, histidine decarboxylase; GABA, γ‐aminobutyric‐acid; GAD, glutamic‐acid‐decarboxylase; SCFAs, short‐chain fatty acids; AHR, aryl hydrocarbon receptor; NMDAR, N‐methyl‐d‐aspartate receptor; nAChRs, nicotinic acetylcholine receptors; GPR, G‐protein‐coupled receptor; TRP channels, transient receptor potential channels; TRPV1, transient receptor potential vanilloid 1; CGRP, calcitonin gene‐related peptide; IL, interleukin; HDAC, histone deacetylase; NO, nitric oxide; PKA, protein kinase A; PKC, protein kinase C; MAPK, mitogen‐activated protein kinase.

Microbiota‐derived metabolites play a crucial role as significant mediators that activate nociceptors, providing a direct mechanism for pain. The precise mechanisms by which microbial products modulate pain perception are summarized as follows (Figure 3).

#### 4.1.1 Tryptophan‐kynurenine pathway

The tryptophan (Trp)‐kynurenine (Kyn) pathway is closely associated with pain sensitivity. As an essential amino acid, Trp plays a pivotal role in maintaining gut amino acid metabolism and microbiota homeostasis [21]. Most Trp is obtained from the diet, and the gut microbiota are also important producers of Trp [22]. Approximately 95% of Trp undergoes catabolism to Kyn via catalysis by the key enzyme indoleamine 2,3‐dioxygenase (IDO) [23]. Elevated expression of IDO has been observed in serum samples from patients with IBD and IBS, accompanied by decreased levels of Trp and increased levels of Kyn [24, 25]. Moreover, the serum Kyn/Trp ratio shows significant elevation in patients suffering from various pain conditions [26, 27]. Correlation analysis further suggests that the Kyn/Trp ratio can serve as a potential marker for assessing IBD activity [28, 29]. In terms of mechanism, Kyn mainly promotes the secretion of several algogenic cytokines, such as GM‐CSF, IL‐37, and TNF‐α [26, 27, 30].

The gut microbiota, such as *Escherichia coli*, is metabolically able to convert Kyn to kynurenic acid (KynA) [31]. As a competitive antagonist of N‐methyl‐d‐aspartate (NMDA) receptors, KynA alleviates migraine *in vivo* by decreasing the expression of transient receptor potential vanilloid 1 (TRPV1) in the DRG and the release of calcitonin gene‐related peptide (CGRP) [32]. KynA also inhibits MAPK/NF‐κB signaling in mouse models of trigeminal neuralgia, with consequences for decreased secretion of pain‐inducing cytokines [33]. Furthermore, KynA noncompetitively suppresses α7 nicotinic acetylcholine receptors (nAChRs). Inhibitors of α7 nAChRs exert analgesic effects in vivo, implying the therapeutic potential of KynA in neuropathic pain [34]. However, the effects of KynA on pain are not unilateral. KynA activates GPR35, leading to NLRP3 inflammasome degradation and decreased IL‐1β production. The susceptibility to colitis is subsequently enhanced [35]. A recent study demonstrated that microbial communities involved in Trp metabolism were dysregulated in children with migraine, with significantly decreased KynA and increased quinolinic acid (QA) in plasma. Gut dysbiosis is characterized by elevated abundances of *Bacteroides* and *Sutterella*, while the proliferation of *Faecalibacterium* and *Bifidobacterium* is greatly suppressed [36]. The differential role of KynA in pain regulation may be attributed to its distinct site‐specific actions, acting as a centrally mediated analgesic while exerting an algogenic effect in the gut. QA is another downstream metabolite of Kyn and is known to be an underlying pain‐inducing factor. QA activates NMDARs to induce inward currents and exacerbates neuropathic pain in vivo [37, 38].

#### 4.1.2 Indoles and indole derivatives

Indoles and indole derivatives, ligands of aryl hydrocarbon receptor (AHR), contribute to maintaining intestinal homeostasis. Previous studies have shown that IDO, which potentiates the conversion of Trp to Kyn, is highly expressed in patients with IBD, competitively mitigating indole derivative synthesis and aggravating hyperalgesia [39]. The gut microbiota can convert indole and indole derivatives from Trp, such as, *Peptostreptococcus russellii*, and *Lactobacillus* genera [40, 41]. Some mediators are essential for indole production in the gut microbiota. For instance, *Card9* is a famous IBD susceptibility gene that encodes an important intracellular signaling protein in the host. With the help of CARD9, gut microbiota homeostasis and Trp metabolic stability are maintained. The production of indole derivatives by the gut microbiota is decreased, and the activation of AHR in the colon is blocked in *Card9*‐deficient mice, with consequences for the aggravation of colitis. Indoles and indole derivatives can promote the production of IL‐22, a crucial cytokine that maintains gut homeostasis and alleviates colitis symptoms, by innate lymphoid cells and T cells [41, 42]. These data are in line with cross‐sectional studies reporting that decreased expression of indole derivatives and AHR leads to lower concentrations of IL‐22 in patients with IBD [43]. Recently, a new anti‐inflammatory mechanism of AHR was discovered in zebrafish and mouse models of IBD. AHR hyperactivation by an indole derivative can inhibit the production of the NF‐κB‐driven transcription factor C/EBPβ. The promotion of proinflammatory factor synthesis by C/EBPβ is diminished, so the release of proinflammatory factors is subsequently reduced in T cells and dendritic cells [44]. Indoles and indole derivatives derived from the microbiota may exert anti‐inflammatory and analgesic effects through this mechanism.

#### 4.1.3 Serotonin

Serotonin (5‐HT) is another mediator synthesized by Trp and is involved in a variety of physiological activities, such as vasoreactivity, cell growth, and intestinal motility. The regulatory functions of 5‐HT rely on the categories of 5‐HT receptors, which include 15 subtypes, and determine the algogenic or analgesic roles of 5‐HT [45]. For example, when binding to the 5‐HT_7_ receptor, 5‐HT has a proinflammatory effect and exacerbates colitis [46]. In contrast, activation of the 5‐HT_4_ receptor facilitates wound healing and reduces oxidative stress‐induced apoptosis in vivo, thereby diminishing hyperalgesia [47]. It is difficult to resumptively define 5‐HT as either an analgesic or an algogenic substance. Compared to the physiological situation, 5‐HT levels in the gut vary among different clinical specimens or animal models of inflammatory pain [48]. 5‐HT is synthesized mainly by enterochromaffin cells (ECs) via the enzyme tryptophan hydroxylase 1 (TPH1) [49]. The gut microbiota and its metabolites, such as short‐chain fatty acids (SCFAs) and deoxycholic acid, increase the activity of TPH1 to promote 5‐HT biosynthesis [50, 51]. Furthermore, some bacteria, such as *Corynebacterium* spp., *Streptococcus* spp., and *Escherichia coli*, can decarboxylate Trp to synthesize 5‐HT, a mode of synthesis independent of TPH1 [50]. The bacteria are critical for regulating serotonin‐related pain. Clostridiales dysbiosis, observed in patients with IBS, is accountable for the dysregulation of the 5‐HT system. This dysbiosis disrupts the normal brain‐gut microbiome axis in IBS patients, ultimately resulting in altered pain perception [52].

#### 4.1.4 Histamine

Histamine was first isolated from *Claviceps purpurea* in 1910 [53], and mast cells were subsequently identified as important producers of histamine. Histamine is generated from l‐histidine via catalysis by the enzyme histidine decarboxylase (HDC). As a crucial signaling molecule, histamine is implicated in physiological processes such as immune responses, allergic reactions, and neural messages [54]. Histamine receptors include H1Rs, H2Rs, H3Rs, and H4Rs. Activation of H1Rs and H4Rs may exacerbate intestinal inflammation and abdominal pain in vivo [55]. Moreover, histamine can further sensitize TRP channels distributed in nociceptor neurons (e.g., TRPV1, TRPA1, TRPV4), which exacerbates hyperalgesia in models of IBS [56, 57]. Multiple gut bacterial genera have been identified as prolific histamine producers [58]. *Klebsiella aerogenes* carrying a highly mutated *Hdc* gene can robustly produce histamine, which subsequently induces mast cells to release more histamine via their H4Rs. Bacterial histamine also directly causes visceral hypersensitivity via the H4Rs in colonic epithelial cells, which is one reason for abdominal pain in individuals with IBS [59]. Although not proven to be a direct producer of histamine, fungal disruption, characterized by decreased Shannon indices, promotes histamine release from mast cells by activating the Dectin‐1/spleen‐associated tyrosine kinase signaling pathway. This mechanism also serves as an important reason for visceral hypersensitivity in patients with IBS [17]. However, there are receptor type‐specific effects of histamine. Histamine produced by *Lactobacillus reuteri* activates H2Rs and inhibits the production of IL‐6 and TNF‐α, with consequences for colitis amelioration [60, 61].

#### 4.1.5 Glutamate

Glutamate stands as a vital excitatory neurotransmitter in intricate neural communication. As the major mediator of glutamate‐related pain, the NMDA receptor is activated by glutamate to facilitate the transmission of nociceptive signals [4]. When subjected to traumatic stimuli, peripheral nerve terminals release glutamate, which sensitizes nociceptors in afferent neurons, characterized by enlargement of receptive fields, decline in excitability thresholds and prolongation of depolarization [62]. Gut microbiota (e.g., *Peptostreptococcus elsdenii* and *Selenomonas ruminantium*), potent synthesizers of glutamate, hold pivotal importance in its utilization [63, 64]. Individuals with fibromyalgia exhibit elevated serum glutamate concentrations and reduced gut microbial diversity, particularly declines in *Bifidobacterium* and *Eubacterium* genera [65, 66].

#### 4.1.6 γ‐aminobutyric‐acid

γ‐aminobutyric‐acid (GABA) is synthesized via the decarboxylation of glutamate, catalyzed by the enzyme glutamic‐acid‐decarboxylase (GAD). As the primary inhibitory neurotransmitter, GABA facilitates potassium ion outflux to induce hyperpolarization in postsynaptic cells and decrease neuronal signal excitability [67]. Increased local concentrations of GABA typically indicate pain alleviation. Many bacteria, such as *Bacteroides*, *Parabacteroides*, and *Escherichia* species, are important producers of GABA [68]. Reduced abundance of *Ligilactobacillus murinus* accompanied by decreased GABA in feces promotes visceral hypersensitivity in vivo [69]. Conversely, *Bifidobacterium* and *Lactococcus lactis* NCDO2118 can metabolize glutamate to GABA, achieving visceral hypersensitivity mitigation in vivo [65, 70, 71].

#### 4.1.7 SCFAs

SCFAs have undoubtedly been a hot topic of research on the mechanisms underlying pain modulation in recent years. SCFAs are synthesized by the gut microbiota using undigested carbohydrates enriched in the colon, which are preferred energy substrates for colonocytes. They mainly consist of acetate, propionate, and butyrate [72]. The functions of SCFAs in pain modulation are complex and cannot be described simply as either promoting or inhibiting effects.

On the one hand, SCFAs have anti‐inflammatory and analgesic properties. Decreased SCFA levels in the gut are associated with different disease‐associated pain conditions, such as chronic prostatitis/chronic pelvic pain syndrome (CP/CPPS), and temporomandibular disorders (TMDs) [73, 74]. There are multiple mechanisms involved in pain alleviation by SCFAs. GPR41 and GPR43 are GPCRs, and they can be efficiently activated by SCFAs. The recruitment of leukocytes and activation of effector T cells are subsequently promoted through the Erk1/2 signaling pathway. These mechanisms underpin immune homeostasis and prevent excessive inflammatory responses [75]. Additionally, activated GPR41 by SCFAs inhibits histone deacetylase (HDAC) to increase the expression of AHR and HIFα, thereby promoting the release of IL‐22 by lymphocytes [76]. Downregulated HDAC3 expression also suppresses the secretion of proinflammatory mediators, such as IL‐6, IL‐12, and nitric oxide [77]. Notably, dysregulation of the NLRP3 inflammasome and IL‐18 is involved in the pathogenesis of various kinds of pain [78], but they are also essential for maintaining gut epithelial integrity and mitigating visceral hypersensitivity induced by colitis [79]. SCFAs can bind to GRP43 to activate the NLRP3 inflammasome and subsequently promote the production of IL‐18, which is critical for preventing colitis [79, 80]. Furthermore, SCFAs decrease the expression of TRPV1 in DRGs and the release of CGRP in rat models of osteoarthritis, accompanied by decreased TNF‐α, IL‐1β, and CCL2 [81].

SCFAs also act as inducers of hyperalgesia, which is linked to many diseases. The levels of microbiota‐derived SCFAs have been shown to increase in the serum of patients with fibromyalgia and in the feces of mouse models of postinfectious IBS [82, 83]. One cross‐sectional study indicated that increased SCFA levels in feces are related to abdominal pain in IBS patients, accompanied by an increase in the abundance of *Veillonella* and *Lactobacillus*, which are known to produce SCFAs [84]. Mechanically, microglia exhibit both M1 phenotype (proinflammatory) and M2 phenotype (anti‐inflammatory), and SCFAs promote the polarization of microglia in a proinflammatory direction to aggravate pain [85, 86]. Furthermore, SCFAs increase TRPV1 expression in nociceptor neurons, increasing nociceptor sensitivity in rodent models of colitis [87, 88]. This is contrary to the findings of the rat models of osteoarthritis described above, suggesting that there are different results from different experimental modeling methods. Overall, the intricate ramifications of SCFAs epitomize the multifaceted intricacies underlying pain mechanisms.

### 4.2 Immune system‐dependent indirect mechanisms

**Figure 4:**
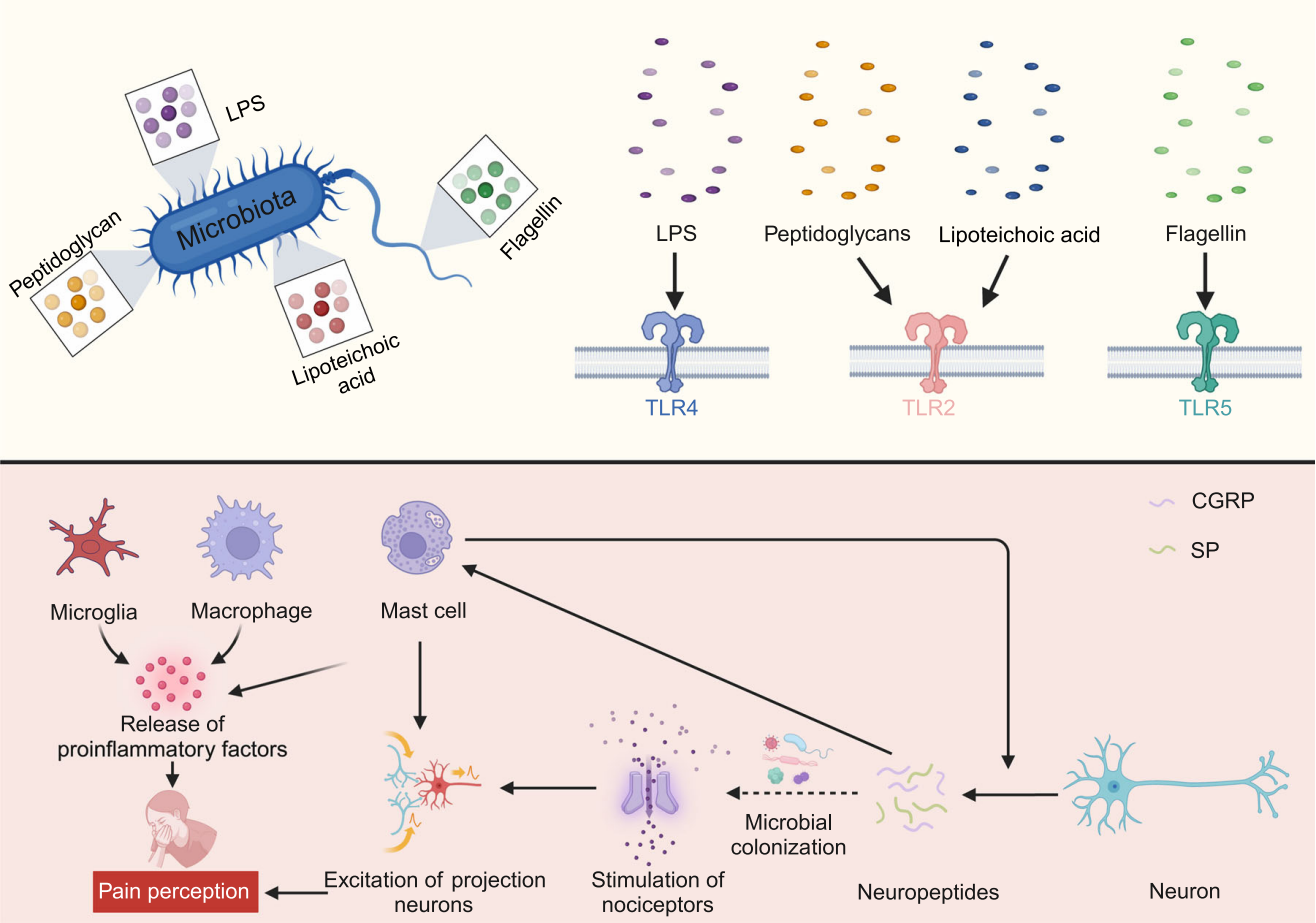
Schematic illustration of the indirect mechanisms by which microbiota modulates pain sensation. The bacterial components including LPS, peptidoglycans, lipoteichoic acid, and flagellin can be recognized by TLRs. Activation of TLRs in macrophages, microglial cells, and mastocytes promote the release of proinflammatory factors, which subsequently induce pain hypersensitivity. Furthermore, mastocytes can directly enhance the excitation of projection neuron. The positive feedback between mastocytes and neurons via two neuropeptides, CGRP and SP, also contributes to pain development. CGRP, calcitonin gene‐related peptide; LPS, lipopolysaccharide; SP, substance P; TLR, toll‐like receptor.

Microbiota dysbiosis prompts immune cells to release regulators of host immunity, such as inflammatory factors and chemokines, which subsequently act on nociceptor neurons or promote neuron‐immune crosstalk. The regulation of pain by the microbiota through the immune system is an indirect mechanism (Figure 4).

#### 4.2.1 Pathogen‐associated molecular patterns (PAMPs)

PAMPs are structurally constant and evolutionarily conserved molecular structures specific to the microbiota. PAMPs can be divided into bacterial surface components and nuclear components. The bacterial surface components mainly include LPS, peptidoglycans, lipoteichoic acid (LTA), and flagellin, and the nuclear components mainly consist of CpGDNA, ssRNA, and dsRNA. Pattern recognition receptors (PRRs), such as Toll‐like receptors (TLRs), are present in innate immune cells and recognize PAMPs [89]. The interaction of PRRs and PAMPs promotes the nucleation of the NLRP3 inflammasome, the critical process of inflammasome activation [90]. The activation of the NLRP3 inflammasome by PAMPs such as LPS, peptidoglycan, and nigericin (*Streptomyces hygroscopicus*) promotes the production of mature IL‐1β [91]. The NLRP3 inflammasome and IL‐1β are positively associated with pain perception [92]. IL‐1β promotes the phosphorylation of the Nav1.8 sodium channel by p38 MAPK on nociceptor neurons to induce action potentials and directly trigger pain sensation [93]. IL‐1β can also sensitize nociceptors or enhance neuronal‐glial interactions, causing peripheral or central sensitization and indirectly triggering hyperalgesia [94, 95]. Furthermore, PAMPs facilitate the release of other proinflammatory cytokines, including TNF‐α and IL‐6, as well as chemokines, such as CCL2 and CXCL1 [96]. Dysregulation of proinflammatory factors sensitizes nociceptor neurons to noxious stimuli and increases action potential frequencies, promoting the onset and progression of pain [97].

#### 4.2.2 Neuropeptides

Neuropeptides, such as substance P (SP) and CGRP, are endogenous neuroactive substances released by nociceptor neurons when exposed to pathogens or inflammatory mediators produced by the immune system. Activated neuropeptides subsequently regulate the function of the immune system [97]. CGRP levels are positively correlated with pain severity in various diseases [98]. A recent study revealed that nociceptor neurons produce more CGRP in germ‐free mice, accompanied by greater visceral sensitivity when exposed to harmful stimuli [99]. There is also increased colonic SP in mice suffering from visceral hypersensitivity with nonabsorbable antibiotic‐induced flora dysbiosis [100]. The commensal microbes contribute to the production of CGRP by nociceptor neurons at stable levels [20], suggesting that microbial intervention may be efficacious in mitigating visceral hypersensitivity. However, neuropeptides do not always act as pain inducers. SP and CGRP are essential for maintaining organismal homeostasis. Chemically silenced TRPV1^+^ nociceptors fail to produce SP, resulting in microbiota dysbiosis and increased severity in a dextran sodium sulfate‐induced mouse model of colitis [19]. Similar results of bacterial disruption were also observed in a model with genetic deletion of nociceptors [20]. In addition, CGRP can increase mucin secretion in goblet cells to protect the intestinal mucus layer via the CGRP‐Ramp1 signaling pathway [20]. The density of microfold cells and the immune response are also regulated by CGRP to safeguard the intestine against bacterial infections (e.g., *Salmonella enterica* serovar Typhimurium, *Candida albicans*, and *Staphylococcus aureus*) [18, 101]. Pain is the main symptom of colitis but induces the secretion of neuropeptides by nociceptor neurons, which is a protective mechanism of the host. The use of analgesic therapy without a destination may not be an appropriate way to treat this disease.

#### 4.2.3 Microglia

Microglia are innate immune cells responsible for eliminating abnormal molecules, cells, and pathogens in the central nervous system (CNS), orchestrating tissue homeostasis maintenance and protection [102]. In addition to immune‐related capabilities, microglia play an important role in pathological pain perception, as they can rapidly respond to damage to the spinal cord, peripheral nerves, tissues, and CNS [103]. The gut microbiota integrates gut‐brain communication via vagal transmission and the circulatory system, which are essential for maintaining microglial function [104]. The mTOR signaling pathway regulates cell survival, growth, and proliferation. SCFAs from the gut microbiota suppress DNA damage‐inducible transcript 4, thereby counteracting its inhibitory effects on mTOR signaling. Microglia exhibit global defects in the absence of microbiota‐derived SCFAs [102, 105]. LPS produced by *Klebsiella oxytoca* has been shown to activate microglia by binding to TLR4. Activated microglia secrete proinflammatory factors, including IL‐6, IL‐1β, and TNF‐α [106], which can sensitize nociceptor neurons and induce hyperalgesia [107, 108].

#### 4.2.4 Mast cells

Mast cells are also key to microbiota‐mediated pain. Mast cells are located in the proximity of afferent nerves innervating the meninges, visceral organs, and periphery [109]. The algogenic effect of mast cells relies on vicious interactions with peripheral nerve sensitization. Nociceptor neurons release neuropeptides (e.g., SP and CGRP) and neurotransmitters (e.g., TNF‐α and the IL family) to trigger mast cell degranulation [109, 110]. Hyperactivated mast cells then produce algogenic substances, such as neuropeptides and histamine, which in turn exacerbate the hypersensitivity of nociceptor neurons [111]. The microbiota is closely related to mast cell activation. Luminal endotoxins (e.g., LPS and trypsin) stimulate mast cells to secrete prostaglandin E2, downregulating serotonin reuptake transporter in the gut epithelium to enrich 5‐HT in vivo. This mechanism can partially explain visceral hypersensitivity in patients with IBS [112]. A recent study revealed gut microbiota dysbiosis in mouse models of IBS, which is characterized by increased potentially pathogenic genera, including *Escherichia‐Shigella* spp. and *Eggerthella* spp., accompanied by decreased beneficial *Akkermansia* spp. The disrupted bacteria penetrate the colonic mucus layer to the lamina propria, where bacterial displacement is sensed by mast cells through TLR4 and H4Rs. This interaction process subsequently promotes the secretion of algogenic mediators, including tryptase, chymase, and histamine [113]. Bacterial histamine induces visceral hypersensitivity via the H4Rs [59]. When binding to H2Rs, histamine can augment the sensitivity of nociceptor neurons to harmful stimuli by increasing Nav1.8 channel expression [114]. In addition, Mast cells are important producers of TNF‐α when exposed to LPS [115]. TNF‐α sensitizes nociceptor neurons by upregulating the expression of TRPV1 [116]. TNF‐α also rapidly modulates pain through p38 MAPK‐mediated phosphorylation of Nav1.8 [116, 117]. Fungal dysbiosis‐associated antigenic translocation also stimulates mast cells. β‐glucans, components of the fungal cell wall, induce mast cell degranulation to release histamine via the dectin‐1/Syk pathway, partially explaining why patients with IBS have visceral hypersensitivity. Fungicides have been demonstrated to ameliorate visceral hypersensitivity *in vivo* [17]. A poor diet also contributes to mast cell‐related hyperalgesia. Fermentable oligosaccharides, disaccharides, monosaccharides, and polyols (FODMAPs) are enriched in histaminic acid, which can be decarboxylated to histamine in *Klebsiella aerogenes* [59]. FODMAPs can increase bacterial displacement‐related LPS, which activates mast cells via TLR4 and exacerbates visceral hypersensitivity in models of IBS [118, 119].

### 4.3 Direct stimulation of nociceptors by microbial components

**Figure 5:**
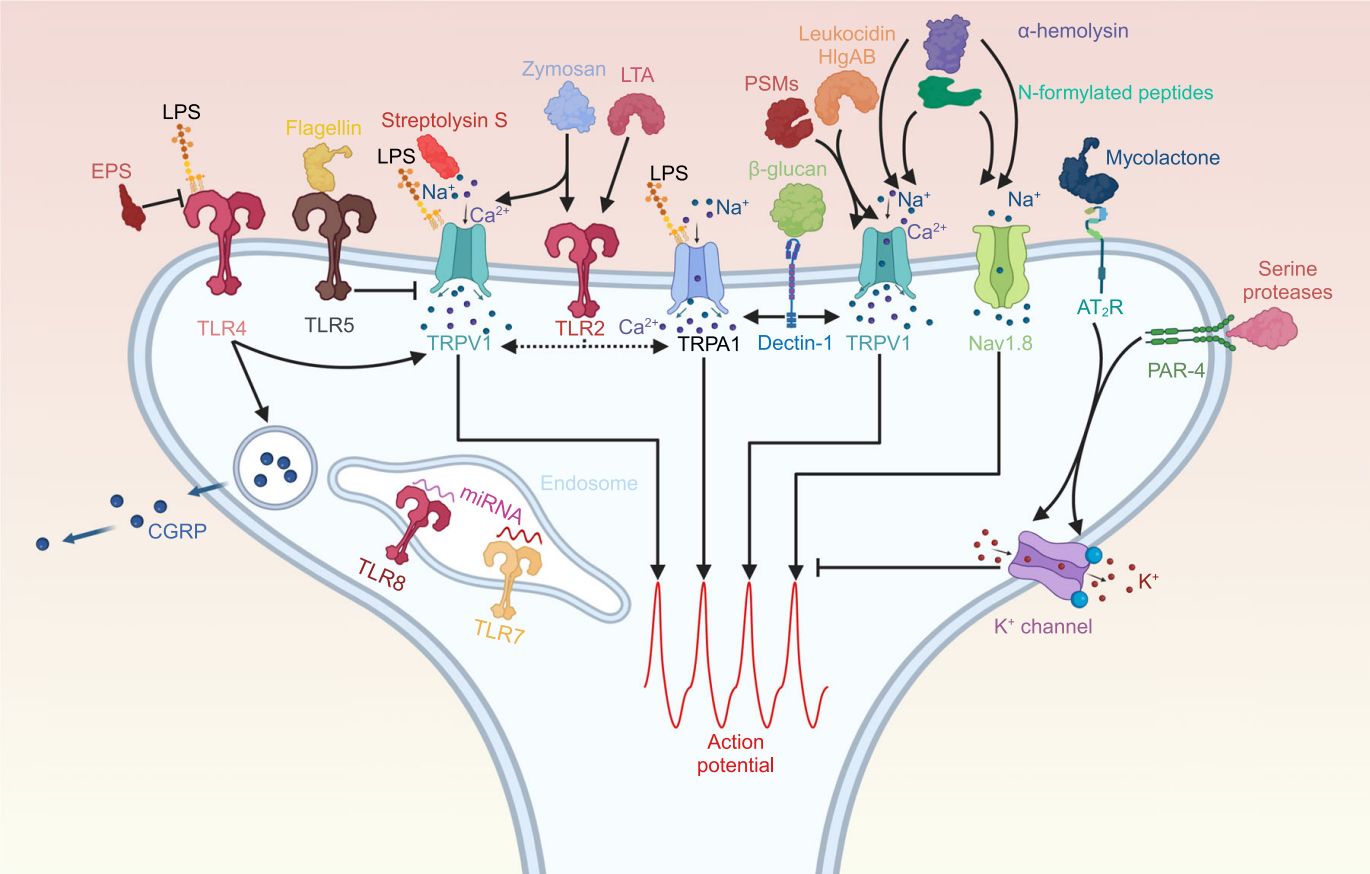
Schematic illustration of direct stimulation of nociceptors by microbiota or their components. Multiple TLRs are localized to various regions and can recognize a range of ligands. TLR7 and TLR8 are identified as receptors for endogenous microRNAs within endosomes. LPS stimulates TLR4 to induce CGRP release and TRPV1 sensitization, while EPS cloaks LPS from TLR4 detection. Flagellin activates TLR5 and subsequently inhibits Na^+^ and Ca^2+^ enter the TRPV1. β‐glucan induces the activation of TRPV1 and TRPA1 by dentin‐1 to cause allodynia. Zymosan stimulates TLR2 and then may activate TRPV1 and TRPA1 to induce cation influx and pain sensation, with dashed arrows indicating mechanisms that are speculative and require further investigations. LPS, streptolysin S, zymosan, PSMs, leukocidin HlgAB can directly activate TRPV1 channel. N‐formylated peptides and α‐hemolysin activate TRPV1 and Nav1.8 to trigger cation influx and action potentials. Increased cation influx by activated TRP and Nav channels subsequently elicits action potentials. Interestingly, some bacterial toxins and enzymes exert analgesic effects. Mycolactone by the AT_2_ receptor and commensal bacterial serine proteases by PAR‐4 promote potassium efflux and neuronal hyperpolarization to ameliorate pain sensation. AT_2_R, type 2 angiotensin II receptor; CGRP, calcitonin gene‐related peptide; EPS, exopolysaccharide; LPS, lipopolysaccharide; LTA, lipoteichoic acid; PSMs, Phenol‐soluble modulins; PAR‐4, proteinase‐activated receptor‐4; TLR, toll‐like receptor; TRP channels, transient receptor potential channels.

Microbes or their components also exert an influence on nociception through direct interactions with nociceptor neurons (Figure 5). This intricate mechanism enables organisms to promptly respond to noxious stimuli for self‐preservation.

TLRs, distributed in nociceptors for PAMPs recognition, are the predominant PRRs that sense microbiota components. Multiple TLRs (e.g., TLR2, TLR3, TLR4, and TLR5) exist in distinct regions and recognize different ligands. For instance, TLR2 detects LTA, TLR4 senses LPS, while TLR7 and TLR8 are activated by endogenous miRNAs [96, 120]. The adaptor protein MyD88, expressed in nociceptors, is required for transcriptional activity of TLRs [96, 121]. Stimulated TLRs can activate MyD88 and subsequently induce immune response by NF‐κB and MAPK signaling pathways in models of neuropathic pain [121], a mechanism by which nociceptors modulate immunity.

TLR4 is a prototypical receptor mediating microbiota‐induced nociception, identified in trigeminal and DRG afferents. Bacterial LPS stimulates TLR4 to promote inward currents, intracellular calcium accumulation, and CGRP release, sensitizing TRPV1 and triggering inflammatory hyperalgesia in vivo [122]. Notably, exopolysaccharide (EPS) derived from Gram‐negative bacteria (e.g., *Pseudomonas aeruginosa*) cloaks LPS from TLR4 detection in TRPV1^+^ nociceptors, mitigating acute host responses. EPS disrupts complement and immune cell phagocytosis, an immune evasion mechanism, which constitutes one of the reasons why clinically asymptomatic patients have persistent inflammation and histological destruction in pathology [123]. Such immune escape strategy underscores that clinical pain inadequately reflects disease status, necessitating intervention in pain‐free individuals.

Other TLRs also interact with microbiota. Bacterial surface components LTA and yeast cell wall composition zymosan stimulate TLR2, which then may activate TRPV1 and TRPA1 to induce cation influx and pain sensation in vivo [124]. Although playing an algogenic role, LTA is crucial for enteric nervous system development and function. Multiple intestinal dysfunctions occur with impaired TLR2 signaling process [120]. The function of TLR5 is more complex. Its agonist flagellin exacerbates abdominal pain severity in maternal separation models [125]. However, another study finds that the combination of flagellin and TLR5 augments neuronal uptake of the sodium channel blocker QX‐314 by TRPV1, and the combined application of flagellin and QX‐314 attenuates allodynia by blocking the entry of Na^+^ into TRPV1 in vivo [126]. Different modeling methods and sample sites may account for divergent pain outcomes. Further investigations are warranted to elucidate TLRs contributions to pain regulation.

Synergistic interplay exists between TRP channels and TLRs in pain regulation. Compared to wild‐type mice, TRPV1 activation alone attenuates pain sensation in *Tlr4* knockout mice [127], implying TRP channel dependency on TLRs. However, several studies have found bacterial LPS directly acts on TRP channels, such as TRPV1, TRPV4, and TRPA1, to induce Ca^2+^ influx and action potential, a TLR‐independent mechanism [128, 129]. Beyond LPS, other bacterial products also stimulate TRP channels. *Staphylococcus aureus* infection causes pain and acidosis. Its cellular component, Phenol‐soluble modulins (PSMs) and leukocidin HlgAB, are key molecules in pain modulation. The dysregulated H^+^ stimulates TRPV1 to provoke cation influx and action potential, culminating in hyperalgesia [130]. Similarly, streptolysin S derived from *Streptococcus pyogenes* binds to TRPV1, causing severe hyperalgesia in the host. However, streptolysin S‐induced CGRP secretion inhibits neutrophil function, while *Streptococcus pyogenes* chemokine‐deactivating proteases ScpC and ScpA suppress neutrophil recruitment. These immunosuppressive mechanisms hijack host defense, which brings about the hallmark “pain out of proportion” in patients with necrotizing fasciitis [131]. Fungi also can directly activate nociceptor neurons. Zymosan derived from *Candida albicans* directly stimulates TRPV1^+^ nociceptor neurons to generate allodynia in vivo [132]. Similarly, β‐glucan promotes phosphorylation of PLCγ2 by dectin‐1 in DRGs, inducing the activation of TRPV1 and TRPA1 to cause allodynia in vivo [133].

In addition, other direct pain‐regulatory mechanisms have been observed in several infectious diseases. N‐formylated peptides and α‐hemolysin from *Staphylococcus aureus* activate TRPV1 and Nav1.8 to trigger cation influx and action potentials in nociceptor neurons. Silencing or blocking Nav1.8 alleviates hyperalgesia in vivo [12]. Interestingly, some bacterial toxins and enzymes exert analgesic effects. Mycolactone from *Mycobacterium ulcerans* binds to the Angiotensin II (AT_2_) receptor, which triggers potassium efflux and neuronal hyperpolarization *in vivo*, underlying the painless lesions of Buruli ulcer [134]. Commensal bacterial serine proteases directly act on proteinase‐activated receptor (PAR)‐4, promoting potassium efflux to reduce the excitability of DRG neurons and ameliorate pain sensation in vivo [135]. This suggests that some bacterial products may exert potential analgesic applications in the future.

## 5 CLINICAL SIGNIFICANCE OF MICROBIOTA IN PAIN MANIPULATION

**Figure 6:**
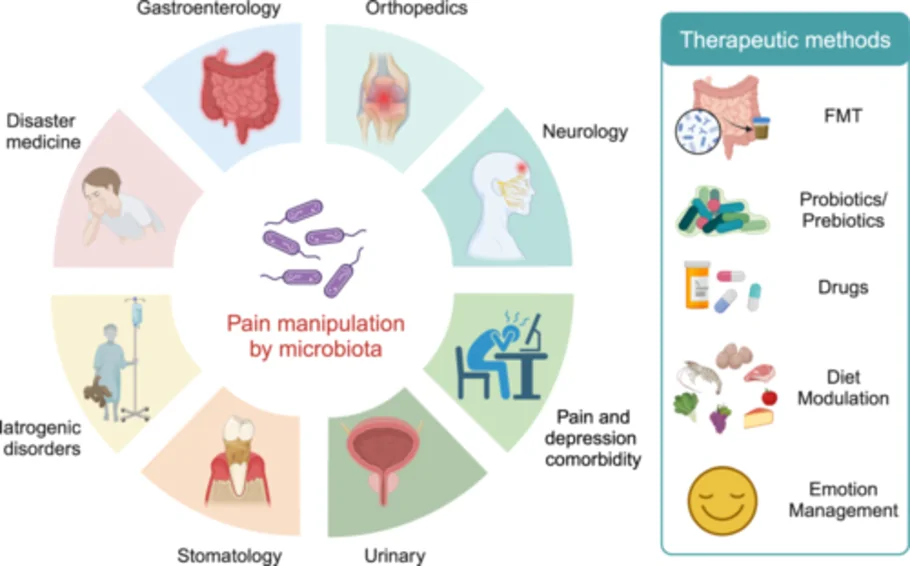
Schematic illustration of current achievements of pain manipulation by microbiota modulation. Accumulating evidence has shown that restoration of microbiota dysbiosis may have potentials in treating diseases mainly in gastroenterology, orthopedics, neurology, urinary, stomatology, iatrogenic disorders, disaster medicine, and comorbidity of pain and depression. The reported therapeutic methods can be concluded into five aspects, including FMT, probiotics/prebiotics, drugs, diet modulation, and emotion management. FMT, fecal microbiota transplantation.

The breadth of research on microbiota and pain has guided the development of related approaches to clinical pain evaluation and analgesics. Investigations into associations between microbiota dysbiosis and pain progression have promoted convenient diagnosis and evaluation of pain (Table 1). Novel interventional regimens have also entered clinical application, including FMT, probiotics/prebiotics supplementation, drugs, dietary modulation, and emotion management (Figure 6). In this section, current achievements are summarized according to clinical specialties to achieve a clear and comprehensive presentation for clinicians.

**Table 1:** The summary of microbiota potentials in diagnosis and evaluation of pain.

Diseases	Sample type	Indicators	Diagnostic potentials	References
IBS	Feces	Genus *Bifidobacterium*	Prediction of sensitivity to FODMAP‐induced IBS symptoms	[136]
IBS	Feces	A panel with 9 bacterial genera	Diagnosis	[137]
IBS	Oral colonizing bacteria	60 otus, 4 genera, 5 families, and 4 orders of bacteria	Diagnosis and severity evaluation	[138]
IBS	Feces	Microbiome and metabolomics	Diagnosis and stratification of children with IBS	[139]
IBS	Feces	*Veillonella* and *Lactobacillus*	Correlation with abdominal pain	[84]
IBS	Feces	*Collinsella aerofaciens*	A predictor of probiotic efficacy	[140]
Pain following radiation therapy for gynecologic cancer	Vaginal colonizing bacteria	Genus *Delftia*	Association with pain following vaginal intercourse	[141]
Knee osteoarthritis	Feces	Genus *Streptococcus*	Osteoarthritis pain severity	[142]
Knee osteoarthritis	Feces	Genus *Streptococcus*	Osteoarthritis pain severity	[143]
Axial spondyloarthritis	Feces	Microbiome	Axial spondyloarthritis pain severity	[144]
Hand osteoarthritis	Feces	Genera *Bilophila*, *Desulfovibrio*, and *Roseburia*	Osteoarthritis pain severity	[145]
Acute pain perception of young healthy male subjects	Feces	Phyla Bacteroidetes and Firmicutes	Correlation with pressure pain threshold	[146]
Small intestinal bacterial overgrowth	Duodenal aspirate	Genera *Klebsiella*, *Escherichia*, *Enterococcus* and *Clostridium*	Correlation with pressure pain threshold	[147]
Caries	Teeth colonizing bacteria	Genera *Gammaproteobacteria*, *Actinomyces*, *Propionibacterium* and *Lactobacillus*	Correlation with toothache	[148]
Postoperative pain of cholecystectomy	Feces	Genus *Proteobacteria*	Prediction of pain and diarrhea occurrence	[149]
Postoperative pain of breast cancer resection	Feces	Genus *Alloprevotella*	Correlation with peak pain during the first post‐operative 24 h	[150]
Postoperative pain of breast cancer resection	Feces	Genera *Ruminococcus*, *Allisonella* and *Acidaminococcus*	Elevation of efficiency of the predictive model based on clinical characteristics	[151]
Fibromyalgia	Feces and blood samples	Genera *Bifidobacterium* and *Eubacterium*	Diagnosis	[66]
Fibromyalgia	Feces	Gut microbiome	Diagnosis	[82]
PTSD	Oral colonizing bacteria	Bacteria *sp_HMT_914*, *332* and *871* and *Noxia*	Correlation with idiopathic pain	[152]
Pelvic pain	Urethra	*Escherichia coli* strain NU14	Correlation with infection‐associated pain	[153]
Buruli ulcer	Skin	*Mycobacterium ulcerans*	Results in painless ulcers	[134]
TMDs	Feces	Bacteroidetes and *Lachnospiraceae*	Correlation with joint pain	[73]

### 5.1 Gastroenterology

The diagnosis and evaluation of IBS pose challenges in clinical practice, primarily relying on exclusionary diagnosis due to the absence of distinctive pathological signs. Considering the significant association between bacteria and IBS progression, a panel consisting of 9 bacterial genera has been developed as a potential noninvasive stool test for diagnosing IBS [137]. Furthermore, leveraging the advantages of machine learning in data processing, combining microbiome and metabolomic biomarkers has demonstrated remarkable performance with an area under the curve (AUC) of 0.93 [139]. Additionally, certain bacteria that utilize fructan as a carbon resource, such as *Bifidobacterium*, can serve as indicators for formulating dietary interventions for individuals with IBS [136]. Interestingly, there is a robust correlation between oral microbiota composition and pain intensity in overweight patients with IBS, suggesting the potential utility of oral microbiome profiling as a hallmark for visceral hypersensitivity [138].

Probiotic supplementation has demonstrated translational value in the therapy of IBS. Administration of *Saccharomyces boulardii* reduces colonic TRPV1 expression and alleviates pain sensation in an IBS model [154]. *Lactobacillus paracasei*, butyrate‐producing *Roseburia hominis*, and pasteurized *Akkermansia muciniphila* attenuate visceral hypersensitivity by mitigating dysfunctions of gut homeostasis, including inflammation, barrier hyperpermeability, and microbiota dysbiosis [100, 155, 156]. Both *Bifidobacterium dentium* and *Lactococcus lactis* possess enzymatic decarboxylation properties for glutamate. The analgesic effectiveness of microbial GABA is observed in models of visceral hypersensitivity [70, 71]. *Lactobacillus plantarum* ameliorates nociceptive perception induced by 5‐HT through downregulation of responses from the hypothalamic‐pituitary‐adrenal axis [157]. The efficacy of probiotic supplementation has also been confirmed through clinical trials, with a primary focus on *Bifidobacterium*. Interestingly, both heat‐treated *Bifidobacterium longum* CECT 7347 and heat‐treated *Bifidobacterium bifidum* MIMBb75 exhibited significant efficacy in relieving abdominal pain. Moreover, the heat‐treated strain displayed enhanced product stability and tolerance [158, 159]. Similarly, in dogs with IBD, although there was no histopathological improvement in inflammation, probiotics partially ameliorated clinical symptoms [160]. In addition to natural probiotics, researchers have explored the potential of bioengineering to harness their advantages. Advances in bioengineering and molecular biology have enabled the construction of a *Lactococcus lactis* strain carrying plasmids for IL‐22 expression, which has demonstrated significant inhibition of IBS symptoms [83]. Similarly, genetically engineered *Escherichia coli* Nissle 1917 overexpressing antioxidant enzymes and EcN@PC–Fe/HA probiotics alleviated colitis and modulated the intestinal microbiome in a mouse model of IBD [161, 162]. However, a systematic review indicates that the efficacy of commonly used probiotics in relieving pain among patients with IBS remains unverified [163]. This discrepancy suggests that currently reported regimens may lack universal effectiveness, and further exploration is needed for probiotic strategies.

Due to the limitations of probiotic supplementation, whole microbiome transplants from healthy individuals have been utilized in the treatment of IBS. Notably, oral consumption of fecal juice was historically employed for treating patients with food poisoning or diarrhea 1700 years ago [6]. Clinical trials validate the efficacy of FMT. Administration of freshly processed feces from well‐defined donors significantly improved IBS symptoms in recipients without any significant adverse events observed. Subsequently, analysis using 16S rRNA gene sequencing revealed a decrease in recipient dysbiosis index, indicating a reduction in gut microbial dysbiosis [164, 165]. Moreover, two clinical trials exhibited improvements in colitis symptoms among patients with IBD following FMT treatment [166, 167]. Repeated FMT treatments can compensate for the unsustainable curative effects achieved through a single intervention. The effectiveness and safety of FMT via different delivery routes, such as nasal feeding and gastroscope have also been confirmed [164, 168], thereby facilitating clinical translation efforts related to FMT.

Diet management is an effective strategy for pain relief in patients with IBS. Restricting the intake of short‐chain fermentable carbohydrates, including FODMAPs, is recommended for managing IBS. A high‐FODMAP diet promotes the growth of gram‐negative bacteria in the gut and increases local concentrations of LPS, thereby contributing to the development of visceral hypersensitivity [118]. Fermentation of FODMAPs leads to increased water and gas volumes in the intestinal cavity, further exacerbating pain perception in individuals with visceral hypersensitivity [169]. The microbial communities associated with immune activation are also reduced after a low‐FODMAP diet [170], and the accumulation of mastocytes and visceral hypersensitivity are attenuated [59]. A research showed that a low‐FODMAP diet can downregulate the abundances of *Klebsiella aerogenes in vivo*, a major producer of histamine and carbohydrate‐fermenting bacterial species [59]. Clinical trials have also confirmed the efficacy of a low‐FODMAP diet in alleviating functional gastrointestinal symptoms, such as abdominal pain, in patients with IBS. Furthermore, this dietary intervention resulted in decreased urinary histamine levels and increased richness and diversity of *Actinobacteria* in fecal samples from these patients [171, 172].

Other non‐bacterial microorganisms also play a significant role in the pathogenesis of IBS. A recent study presented a cultivated gut fungi catalog and analyzed over 11,000 fecal metagenomes, which identified associations between gut mycobiome characteristics and common diseases, such as IBD [173]. Similarly, noticeable disparities in mycobiome diversity and stability have been observed between IBS patients and healthy controls. Fungal dysbiosis contributes to mucosal immune activation and nociceptor sensitization [17]. Particulate β‐glucan can be considered an immunoregulator that induces mastocyte degranulation and histamine secretion against fungal antigens. Administration of β‐glucan effectively inhibits visceral hypersensitivity [17]. Miltefosine, an antibiotic drug used for leishmaniasis, alleviates abdominal pain associated with IBS by rectifying fungal symbiosis and reducing TRPV1 activity [174].

### 5.2 Orthopedics

Musculoskeletal diseases impose significant health burdens. Large‐scale cohort studies have demonstrated that the composition of gut microbiome, particularly *Streptococcus*, *Roseburia*, *Bilophila*, and *Desulfovibrio*, is associated with pain related to osteoarthritis [143, 145]. Oral administration of *Lactobacillus acidophilus* effectively attenuates osteoarthritis‐induced pain through nociceptor desensitization, reduction of spinal glial activities, and suppression of inflammation [175]. A randomized controlled trial (RCT) demonstrated that prebiotics can reduce trunk fat accumulation, thereby mitigating obesity‐related knee osteoarthritis, highlighting their potential therapeutic value in treating this condition [176]. Supplementation with *Lactobacillus paracasei* S16 decreases serum glutamate levels and mitigates inflammatory pain associated with lumbar disc herniation *in vivo* [177]. Certain natural compounds and drugs have also revealed their antinociceptive properties. Resveratrol treatment reverses the reduction in SCFAs‐producing gut bacteria (e.g., *Bacteroidetes* and *Lachnospiraceae*) in models of TMDs induced by complete Freund's adjuvant. The integrity of the blood‐brain barrier and microglial overactivation is subsequently restored as a result of reduced aggravation from the microbiota, leading to the inhibition of cerebral inflammatory responses [73]. Additionally, resveratrol has the ability to promote microglial M1‐to‐M2 polarization in mouse models of LPS‐induced neuroinflammation, thus exerting neuroprotective effects [178].

Fibromyalgia is a common chronic widespread musculoskeletal pain disorder. The specific pathogenesis and radical therapeutics are still not clearly understood. The latest research on microbiota has brought new hopes of fibromyalgia diagnosis and treatment, as there are typical differences in microbiota profiles between patients with fibromyalgia and healthy controls [179]. Diagnostic models based on microbiome and metabolome data have been developed, with the highest AUC value reaching 0.9, which significantly surpasses currently employed methods [66, 82, 180]. The probiotic *Bifidobacterium adolescentis* IPLA60004 has the ability to alleviate pain in fibromyalgia, possibly by reducing serum glutamate levels [65]. A Khorasan wheat replacement diet improves fibromyalgia symptoms partly through increasing the production of SCFAs generated from microbiota [181]. A RCT also indicated that FMT significantly increased 5‐HT and GABA while decreasing glutamate levels in fibromyalgia patients, with obvious improvements in pain index and symptom severity [182].

The formation of biofilms is another mechanism underlying orthopedics‐related pain. Biofilm is defined as aggregates of microorganisms in which cells are frequently embedded in a self‐produced matrix of extracellular polymeric substances that are adherent to each other and/or a surface [183]. Prosthetic materials are extensively utilized for the repair of motor system defects. Infection, characterized by biofilm formation on implant surfaces, represents a critical complication following prosthesis implantation [184]. Biofilm‐active antibiotic therapy is associated with lower pain intensity, better motor function, and better outcome [185]. Several studies have focused on enhancing the antimicrobial properties of implants. For instance, Ag nanoparticles were incorporated into Ti/TixN coatings to effectively inhibit growth rates and adhesion of *Escherichia coli* and *Staphylococcus aureus* to the surface while significantly suppressing biofilm formation [186]. Additionally, immersion of a Ti‐6Al‐4V alloy in a Cu(OH)_2_ solution exhibited efficient attenuation of methicillin‐resistant *Staphylococcus aureus* activity and subsequent prevention of biofilm formation [187]. Phage therapy is an ancient but immature technology for resisting against infection, which has been regarded as a breakthrough of undue reliance on antibiotics. With the rapid development of bioengineering, phage therapy gradually steps into clinical validation and application [188]. One case report showed that the phage therapy was conducted to treat a diabetic man with intractable biofilm‐associated prosthetic knee infection. The biofilm biomass was decreased, and local symptoms were successfully alleviated. This study preliminarily exhibits application prospects of phage therapies in mitigating biofilm‐associated pain intensity [189].

### 5.3 Neurology

Due to the nonrenewable characteristics of neurons, neuropathic pain only relies on symptomatic treatment. Microbiota modulation is a promising direction for the development of more effective and accepted regimens. Typical disturbances in the gut microbiota have been detected in neuropathic pain models [190]. The F/B ratio is considered a representative indicator of gut microbiota dysregulation [191]. Oral administration of antibiotics alters gut microbiota composition and alleviates chronic constrictive injury‐induced neuropathic pain. These antibiotic‐induced flora promote an increased proportion of Foxp3^+^ regulatory T cells while decreasing IFN‐γ‐producing Th1 cells [192]. Palmitoylethanolamide, an endocannabinoid‐like lipid mediator, could mitigate neuropathic pain induced by vitamin D deficiency through restoring gut microbiota symbiosis [193]. Emodin and ursolic acid have also been found to relieve neuropathic pain by regulating the gut microbiota and suppressing the inflammatory response [194, 195]. A probiotics regimen combined with adipose‐derived mesenchymal stem cell treatment alleviates chronic neuropathic pain [196], whereas single administration of probiotics presents lackluster performance [197]. Notably, as a comprehensive microbiota intervention strategy, FMT from healthy mice effectively alleviates neuropathic pain in model mice [198, 199].

Microbiota dysbiosis is related to central neurological abnormalities [200], among which migraine is a representative disease [201]. In contrast to the research on neuropathic pain, the achievements of probiotic application are relatively mature. Treatment with propionate and butyrate changes microbiota distributions, restores intestinal integrity, and attenuates hyperalgesia in the nitroglycerin‐induced migraine model [202]. Multispecies probiotic strategies have entered the validation phase through clinical trials, demonstrating promising therapeutic effects. Following several weeks of probiotic intake, there was a reduction in the frequency or severity of migraine attacks [203, 204].

### 5.4 Urinary

Interstitial cystitis/bladder pain syndrome (IC/BPS) is a chronic and debilitating disease with uncertain etiology, characterized by the clinical manifestations of lower urinary tract symptoms and pelvic pain. However, there are significant challenges in both clinical diagnosis and treatment strategies for this condition. In a pilot study investigating the vaginal and urinary microbiomes, no statistically significant differences were observed in microbiota distributions between premenopausal women with IC/BPS and healthy controls [205]. Another clinical investigation conducted by Natalia et al. compared transcriptomics and bladder body/trigonal epithelia microbiome profiles in adolescent female patients with IC/BPS, along with urine microbiome analysis. Distinct transcriptomic variations were identified between bladder body and trigonal epithelia samples. The microbiome data coincide with the previous study, showing no significant alterations in mucosal and urine microbiome [206]. However, a study focusing on the urinary microbiome of male patients reported opposite results. Urinary microbiota diversity is reduced in patients, together with abundances of *Lactobacillus* species. This microbiome disruption is correlated with cytokine levels [207]. Research covering both sexes indicated that four opportunistic pathogen genera, including *Serratia*, *Brevibacterium*, *Porphyromonas*, and *Citrobacter*, might contribute to IC/BPS development [208]. Additionally, a recent two‐sample Mendelian randomization study found that *Butyricimonas* and *Coprococcus* et al. are positively associated with the risk of IC/BPS, whereas *Desulfovibrio piger* et al. may exert a protective effect [209].

Acyloxyacyl hydrolase is a specific lipoidase expressed in immune cells and endothelial cells that participates in the progression of injury and inflammation. Mice with deletion of acyloxyacyl hydrolase will spontaneously develop pelvic pain, accompanied by altered gut microbiome and metabolomics. Further FMT from control mice alleviates pain perception, directly demonstrating the critical role of microbiota in patients with IC/BPS [210]. Mindfulness‐based stress reduction, an approach to emotional management, has been proven to improve symptoms of IC/BPS. Its efficacies are correlated with urinary microbiome signatures [211].

CP/CPPS is another idiopathic urologic disease characterized by repetitive lower abdominal pain. A two‐sample Mendelian randomization analysis revealed that gut genus *Sutterella* and genus *Holdemania* may contribute to an increased risk of CP/CPPS, while phylum Verrucomicrobia and genus *Parasutterella* are inversely associated with the disease [212]. The reduced abundance of *Prevotella* in gut could potentially serve as a diagnostic biomarker for patients with CP/CPPS [213]. Astaxanthin, a natural anti‐inflammatory compound with probiotic properties, has been shown to increase the abundance of gut *Akkermansia muciniphila* to improve pain generated from CP/CPPS *in vivo* [214]. A clinical trial also showed that treatment with levofloxacin combined with *Escherichia coli* Nissle 1917 for 6 months significantly reduced symptom severity scores and recurrence rates compared to levofloxacin alone in CP/CPPS patients [215].

Ureteral stents are widely employed for relieving ureteral obstruction and promoting ureteral healing after surgery. Similar to other implants, this medical device can be colonized with microbiota. The bacterial biofilms are gradually formed, leading to pain sensation and lower urinary tract symptoms [216]. More importantly, biofilms have no typical responses to antibiotics. Therefore, researchers have developed ureteral stents with anti‐microbial properties by the virtue of interdisciplinary technologies. For instance, the effectiveness of a ureteral stent loaded with triclosan has been verified. It significantly reduces bacterial attachment and biofilm formation. Lower intensities of abdominal pain and urethral pain of patients have also been reported [217]. The antibacterial peptide and Ag@graphdiyne nanocomposite have been used to confer the properties of biofilm suppression on ureteral stents [218, 219].

### 5.5 Stomatology

The oral microbiota plays a crucial yet often overlooked role in human health. Disruption of the balance within oral microbiota communities and biofilms has been established as a significant contributor to stomatological diseases [220]. Burning mouth syndrome is characterized by chronic burning sensations in the absence of detectable oral abnormalities. A comparison of oral microbiomes between patients with burning mouth syndrome and healthy controls revealed decreased microbial diversity in patients, along with the identification of specific genera as predominant bacteria [221, 222]. Caries, a common disease causing toothache, is associated with reduced abundances of *Actinomyces* and *Propionibacterium*‐dominant genera found in healthy teeth. Furthermore, an increased abundance of *Lactobacillus* can indicate caries progression [221]. *Lactobacillus paracasei* ET‐22 inhibits the biofilm of the pathogen *Streptococcus mutans*, with consequences for caries amelioration [223]. Additionally, photodynamic therapy has demonstrated effective analgesic effects for patients suffering from oral mucositis by targeting dysbiosis within the oral microbiota [224].

The formation of dental plaque biofilms is recognized as an important contributor of oral diseases. Biofilms are proven to colonize cracks in all teeth [225]. One study investigated the anti‐biofilm effects of hypochlorous acid stabilized with acetic acid through oral rinsing. The results showed that the viability of supra‐ and sub‐gingival community biofilms was significantly reduced after only 5 min of rinsing, without any observed side effects on oral keratinocytes [226]. This novel oral rinsing fluid exhibits remarkable efficacy in eradicating oral biofilms. Phage therapy is also effective in eliminating dental biofilms. A cocktail consisting of 5 bacteriophages targeting *Salmonella typhimurium* can rapidly inhibit biofilm production by this pathogenic bacterium on tooth surfaces, leading to the destruction of its bacterial structure [227]. Furthermore, the threats posed by biofilms on oral implants have received increased attention in recent years. Materials used for orthodontics and dentures have been improved to more effectively suppress bacterial and fungal biofilms, thereby exhibiting desirable efficacy in inhibiting biofilm formation and preventing pain [228, 229].

### 5.6 Iatrogenic disorders

Postoperative pain is a prevalent complication that hampers the therapeutic benefits of surgery. Notably, significant differences were observed in the microbiome profiles of breast cancer survivors with or without postoperative pain. In an animal model, FMT from subjects experiencing postoperative pain resulted in heightened mechanical hyperalgesia, providing evidence for the causal role of gut microbiota in postoperative pain development. Moreover, a predictive model for postoperative pain was constructed based on multiple bacterial families and genera, achieving an AUC value of 0.808 [151]. This study strongly and comprehensively indicates the translational potential in the diagnosis and treatment of gut microbiota. Pain is also the most prevalent symptom following kidney transplantation. Specific taxa with properties regulating intestinal barrier integrity and chronic inflammation, particularly *Akkermansia*, are associated with posttransplant pain [230]. Dysbiosis of gut microbiota contributes to postoperative pain‐induced cognitive dysfunction and delirium, which can be mitigated by additional supplementation with SCFAs [231]. Additionally, surgical sutures are susceptible to infection and biofilm formation, negatively impacting the normal healing process and exacerbating patients' perception of pain [232]. One study developed a novel surgical suture coated with polymerized cyclodextrin that significantly inhibited bacterial colonization for up to 4 weeks while promoting wound healing [233].

Patients with cancers at advanced and late stages possibly undergo different degrees of pain perception. Beneficial gut microbial populations typically decrease with the aggravation of cancer pain symptoms. Changes in *Ruminococcus bromii* in tumors may be an indicator of cancer healing [234]. In women with colorectal cancer, a deficiency in *Carnobacterium maltaromaticum* was observed, while supplementation with *maltaromaticum* colonization increased intestinal vitamin D levels in an estrogen‐dependent manner and proved beneficial in suppressing the progression of colorectal cancer [235]. On the other hand, chemoradiotherapy can induce iatrogenic pain, further compromising the survival time and quality of life for cancer patients. For example, paclitaxel treatment disrupts the symbiosis of gut microbiota by promoting the proliferation of pain‐sensitive microbiota and reducing pain‐resistant microbiota, subsequently leading to neuropathic pain [236]. Administration of antibiotics effectively eliminates irinotecan‐induced microbiota communities, leading to the alleviation of gastrointestinal pain through the inactivation of TLR4‐dependent mechanisms [237]. Dietary therapy, a widely accepted strategy, has demonstrated its potential in treating chemotherapy‐induced pain. Hydrogen‐rich water restores microbiota structures and reduces LPS levels. Inhibition of the LPS‐TLR4 pathway mitigates inflammatory responses and hyperalgesia [238]. Diosgenin, a natural compound, relieves oxaliplatin‐induced pain by modulating the TLR4 pathway and restoring gut microbiota homeostasis [239].

Radiotherapy for gynecologic cancers greatly changes vaginal microbiota signatures without domination by *Lactobacilli* [240], which serves as an indicator and gatekeeper of vaginal homeostasis. Topical estrogen treatment is associated with the stability and resilience of vaginal communities, indicating the putative role of hormonotherapy in radiotherapy‐induced pain management [141].

Opioids are a class of narcotic analgesic medications commonly used for pain management. However, prolonged exposure to opioids can result in the development of drug tolerance and hyperalgesia upon withdrawal. This pathological process is mechanistically associated with alterations in microbiota symbiosis and translocation, leading to changes in metabolic patterns [241, 242]. Antibiotic treatment has been proven to affect neuron activity and brain region functions involved in drug addiction [243], which raises concerns about antibiotic use for patients receiving opioid medication. FMT from healthy controls rescues opioid‐induced gut microbiota dysregulation, which alleviates excessive activation of microglia and opioid‐induced hyperalgesia [241, 244]. Administration of *Lactobacillus reuteri* increases the expression of peripheral opioid receptors [245]. The abovementioned evidence has proven the promising potencies of microbial regulation therapies.

Fungal peritonitis is a severe complication observed in patients with renal failure undergoing peritoneal dialysis. In a retrospective study conducted by Juliana et al., all patients presented with typical abdominal pain, cloudy peritoneal effluent, and received secondary treatment. A total of six fungi were isolated, with *Candida parapsilosis* being the predominant strain. Notably, most strains exhibited high or moderate biofilm production [246]. Another retrospective investigation focused on the association between bacterial biofilms and hernia mesh infection. Infection of the hernia mesh can result in chronic pain, seroma formation, and failure of hernia repair [247]. Among 20 explanted groin hernia meshes analyzed, only one was negative for bacterial biofilms. These findings highlight the potential contribution of bacterial biofilms to mesh complications and chronic pain following hernia repair [248]. The preventive and therapeutic effects of anti‐biofilm therapies should be extensively verified.

### 5.7 Disaster medicine

Gulf War Illness (GWI) is a debilitating condition that imposes significant health burdens on numerous soldiers. Veterans exhibit distinct gut microbiota signatures, with specific bacterial abundances being correlated with psychopathological symptoms [152, 249], thereby enabling soldier deployment and stratification. Notably, there are characteristic reductions in the abundance of SCFA‐producing taxa [249]. Consistent with expectations, supplementation with butyrate enhances gastrointestinal functions and restores metabolic patterns. Furthermore, peripheral expression of TLR4 and TLR5 is diminished [250]. These findings provide indications that the administration of SCFAs may represent an efficacious approach to alleviating GWI‐associated pain. Treatment involving probiotics or antibiotics ameliorates GWI symptoms by rectifying gut microbiota dysbiosis [251, 252]. In addition to war‐induced hyperalgesia, natural disasters such as floods also induce gastrointestinal dysfunctions and microbiota dysbiosis. Supplementation with one kind of probiotic, *Bifidobacterium infantis*, effectively improved the mental health of victims [253].

Chronic wounds impose significant health and economic burdens globally. The presence of microbial colonization and biofilm formation further complicates wound treatment [254]. Some kinds of wound dressings with anti‐biofilm functions have been created. In an early study conducted by Lenselink et al. in 2011, a cohort of eight patients with chronic wounds was treated using a biocellulose dressing loaded with polyhexanide to assess its efficacy. This dressing effectively suppressed biofilm formation and promoted the growth of granulation tissues, leading to the alleviation of pain during dressing changes [255]. In vitro studies demonstrated that a chitosan membrane releasing cis‐2‐decenoic acid and bupivacaine, two antibacterial agents, could inhibit MRSA growth and prevent biofilm formation. Similarly, a wound dressing releasing nitrogen oxides exhibited potential for inhibiting biofilm formation; however, their safety needs to be carefully evaluated through in vivo experiments due to their significant toxicity towards fibroblasts [256, 257]. Gelatin has also been investigated as a drug delivery material for chronic wound healing. Previous studies focused on coating gelatin with polyhexanide/betaine or silver nanoparticles which effectively eliminated biofilms and facilitated wound healing while reducing pain intensity [258, 259]. A new hydrogel encapsulating *Lactobacillus paracasei* TYM202 helps the skin defend itself against foreign pathogens and maintains skin microbiota homeostasis, contributing to wound healing [260]. Furthermore, the therapeutic potential of phages in wound healing has also been explored. Two phages against *Enterococcus* have been identified and used for the eradication of *Enterococcus*‐related biofilms. *In vitro* experiments have validated the efficacy and safety of this combination phage regimen, highlighting their promising applications in combating biofilms in chronic wounds [261].

### 5.8 Pain and depression comorbidity

Depression is a prevalent comorbidity associated with hyperalgesia in various diseases [1]. As a crucial regulator of biological and physiological processes, the microbiota acts as a mediator in the coexistence of pain and depression. However, specific changes in microbial communities and functions are disease‐specific. In patients with IBS, there is an increased relative abundance of *Ruminococcus torques* and decreased relative abundance of *Fusobacterium* in both the caecum and descending colon [262]. The disturbance in Trp/5‐HT metabolism in feces and serum is correlated with depression severity, which is characterized by a significant conversion of Trp metabolism towards the production of Kyn [263]. Besides gut microbiome alterations, other distinctive microbial compositions have shown potential diagnostic value. Vaginal microbiome diversities serve as indicators for vulvodynia‐depression comorbidity. Close correlations between vulvodynia and depression have been observed specifically among patients with low diversities compared to those with high diversities [264]. In female patients with IC/BPS, urine *Lactobacillus acidophilus* abundance is associated with pain severity, while symptoms of IC/BPS also correlate with depression scores. These findings suggest the potential role of urine *Lactobacillus acidophilus* as an indicator for assessing comorbidity between IC/BPS‐related pain and depression [207]. A set of features related to microbiome composition and metabolomics has been identified as potential biomarkers for evaluating the coexistence of autoimmune prostatitis and depression [265].

Several putative mechanisms have been proposed to elucidate the regulatory role of the microbiota in this comorbidity. For instance, dysregulation of vagus nerve functions has been demonstrated to facilitate the development of both pain and depression [266]. Notably, butyrate, a crucial metabolite derived from the microbiota, can directly activate sensory fibers within the vagus nerve [267]. Electro‐acupuncture modulates the abundance of SCFAs‐producing bacteria and effectively treats comorbid chronic pain and depression [268]. Targeting microbiota dysbiosis has ushered in a new era for addressing the comorbidity between pain and depression. Probiotic therapy, particularly oral administration of Golden bifid—a classical probiotic preparation, significantly alleviates abdominal pain and depression symptoms in patients with IBS [269]. The GABA system plays a crucial role in information processing and cognitive functions. *Lactococcus lactis* has been identified to have properties of GABA production [71]. This strain has been recognized as a promising candidate for further clinical investigations aimed at treating the comorbidity of pain and depression. Other studies found that antibiotics and FMT could effectively reverse the comorbidity of pain and depression [199, 270]. In addition to these microbial interventions, gluten‐free diet and the Chinese traditional formula Chang‐Kang‐Fang can effectively correct gut microbiota dysbiosis following IBS and treat comorbidity of visceral hypersensitivity and depression [271, 272]. Moreover, the expression of IDO, an important enzyme in Kyn pathway, in bilateral hippocampus is significantly elevated, accompanied with Kyn/Trp ratio. Silence of *Ido* gene expression or administration with an IDO inhibitor 1‐methyltryptophan effectively ameliorates pain and depression [273]. Targeting drugs against the Kyn pathway may represent a novel strategy for treating this comorbidity.

## 6 NARROWING RESEARCH GAPS WITH POTENTIAL SOLUTIONS

The strength of evidence based on research is insufficient. Current investigations on pain and gut microbiota frequently employ rodents as experimental models. Rodents possess significant physiological differences from humans, particularly in the nervous system [274]. This raises concern regarding the translatability of rodent‐based findings to human subjects. For instance, several studies have demonstrated that supplementation with SCFAs can contribute to pain relief *in vivo*. However, a RCT found that oral administration of *Lactobacillus paracasei*, with the property of producing SCFAs, did not effectively alleviate abdominal pain in patients with IBS [275]. Therefore, it is imperative to establish more precise and realistic experimental models that bridge these gaps. Mammals may be a better alternative, but the ethical issues involving mammals cannot be ignored. Future directions should focus on developing improved non‐invasive experimental techniques and sampling methods derived directly from the human body.

The collected experimental indicators are often questioned for their objectivity. Pain measurement indicators, such as the writhing response, abdominal wall contraction time, and inflammatory factors, though providing some insights, still have a long way to go to show how experimental animals really feel pain [276]. Researchers should develop a more comprehensive and objective pain assessment scale based on IACUC recommendations that incorporates additional behavioral parameters, including excessive sweating, teeth grinding, and aggressive licking of wounds. Moreover, standardized methods for collecting and processing microbiota measurements in animal models are lacking. Microbial sampling primarily relies on feces or intestinal contents taken after execution in experimental models which may not accurately represent the microbial composition across different segments of the gut. However, there are currently limited viable alternatives for sampling [277]. Smart capsules have the potential to become an important tool for non‐invasive monitoring of gut health through sensing operations that can collect tissue and microbial samples [278].

Piercing through 72 transformations with golden eyes. The microbiome exhibits remarkable diversity, varying across organs, individuals, and environments [279]. Even when subjected to identical modeling conditions, significant variations still exist among different rodents [280]. Moreover, due to various limitations, most current studies have fewer than 10 samples per group, which hinders the accuracy of microbiological measurements. Therefore, it is imperative to increase the sample size in experiments to minimize statistical errors. Although experimental techniques such as 16S rRNA sequencing or shotgun metagenomics facilitate microbial analysis, data obtained from a single sampling time point fail to capture the highly variable nature of microbiota under physiological conditions. Sampling at multiple time points is essential to demonstrate the rigor and robustness of the experimental design. Furthermore, numerous studies have observed that pain in various diseases coincides with specific microbial alterations; however, this only suggests a potential correlation between dysregulated microbiota and pain. To establish causality between microbes and pain requires further intervention and rescue experiments.

The role of non‐bacterial and non‐gut microbiota in pain sensation should be given greater consideration. The popularity of 16S rRNA sequencing has greatly facilitated our comprehension of the role of bacteria in pain; however, it is imperative to acknowledge the significant contribution of non‐bacterial microbiota in pain progression (Table 2). The advancements in metagenomic technology have considerably enhanced our investigation into non‐bacterial microbiota. Some studies examine limited numbers of pain‐causing fungal species; however, evidence suggests that variations in gut fungal community are associated with multiple common diseases [173], indicating the potential oversight of many fungal roles. Recently, gut methane‐producing archaea have emerged as potential culprits for abdominal pain, with robust vitality and unique metabolic characteristics [282, 284], highlighting advancements in microbe‐pain studies. Furthermore, investigations into the mechanisms underlying pain should be strengthened. Although *Candida albicans*‐associated pain is commonly believed to be caused by inflammation, it has been discovered that Zymosan can directly stimulate nociceptor neurons and induce pain [132]. Uncovering new mechanisms may lead to novel targets for pain treatment. Studying the impact of gut fungi on gut bacteria also warrants attention. Notably, penicillin, an iconic milestone in modern medicine renowned for its exceptional antibacterial properties, originates from Penicillium. The significance of non‐gut microbes in pain should be accorded greater attention. Microbiota dysbiosis plays a pivotal role in IC/BPS, CP/CPPS, and oral diseases. Addressing microbial disorders in the bladder, vagina, mouth, and other exposed external environments holds substantial therapeutic potential beyond current research alone. Indeed, this cohort of symbiotic microorganisms, though diminutive in stature, merits extensive scrutiny; for size, after all, is no gauge of their substantial role.

**Table 2:** Existing research on non‐bacterial functions in pain.

Microbiota types	Diseases	Research overview	References
Fungal microbiome	IBS	Intestinal fungal dysbiosis contributed to the development of visceral hypersensitivity, and antifungal therapy could reverse IBS‐related symptoms	[17]
Fungal microbiome	IBS	Miltefosine treatment alleviated visceral hypersensitivity partly through regulating gut mycobiome	[174]
Fungal microbiome	IBD	The relationship between intestinal fungal characteristics and IBD is elucidated	[173]
Fungus: Genera *Candida* and *Malassezia*	Interstitial cystitis	Increased abundances of *Candida* and *Malassezia* were associated with more severe symptoms of patients	[281]
Fungus: *Eubacterium* genera	Fibromyalgia	The intestinal *Eubacterium* genera significantly decreased, accompanied by notable changes in serum metabolomics	[66]
Fungus: *Candida albicans*	Skin pain	*Candida albicans*‐derived β‐glucan cause pain depending on Dectin‐1	[133]
Archaea: *Methanobrevibacter smithii*	Abdominal pain	The relative abundance of *M. smithii*, a high methane emitter, was dramatically upregulated	[282]
Archaea: *M. smithii*	IBS	Treatment with modified‐release lovastatin effectively inhibits methane production by *M. smithii* and mitigated IBS progression	[283]
Archaea: *Methanobrevibacter*	IBS and IBD	The lack of *Methanobrevibacter* is linked to gastrointestinal imbalance	[284]
Protozoa: *Giardia lamblia*, *Dientamoeba fragilis*, *Blastocystis hominis*	Recurrent abdominal pain	No characteristic presentation of protozoa profiles was observed between children with recurrent abdominal pain and healthy individuals	[285]

Abbreviation: M. smithii, Methanobrevibacter smithii.

Microbiota regulation therapy is gaining momentum. Several interventions targeting the gut microbiota play a positive role in relieving pain symptoms. (i) Dietary intervention represents a cost‐effective treatment approach that can circumvent potential drug‐related side effects. Several RCTs support the efficacy of a low FODMAP diet in alleviating abdominal pain [172, 287, 288]. However, dietary treatments should be assessed by a registered dietitian using specific protocols tailored to each patient's unique pathophysiological factors to determine appropriate dietary combinations and intervention duration. These behavioral programs may pose challenges in terms of accessibility and adherence [289]. (ii) Probiotics represent an area of scientific interest with commercial potential. These live microorganisms can confer benefits upon the host when present in sufficient quantities [290, 291], and they have exhibited the great potential in pain relief (Table 3). Notably, supplementation with select strains of fungi, such as *Candida metapsilosis* M2006B, has also exhibited beneficial effects for colitis relief *in vivo* [286]. However, uncertainties remain regarding the suitability of specific probiotic strains for different diseases, optimal treatment duration, and dosage requirements [299]. Further research is warranted to establish efficacy since some study results indicate no significant effect of probiotics on pain management [275]. It is not credible to assume a one‐to‐one correspondence between a strain and its function. Due to the high variability of microbiota among different populations, there is currently insufficient high‐quality clinical evidence to support the use of a standardized probiotic regimen [300]. Additionally, potential side effects of probiotics cannot be disregarded, including diarrhea and bloating [301]. iii) FMT may serve as a complementary therapy to probiotics. Microbiota dysbiosis involves changes beyond single species, making FMT potentially more effective in addressing overall microbial imbalances. Clinical trials have demonstrated the efficacy of FMT in correcting microbial disorders, including its potential to ameliorate extra‐intestinal diseases such as Fibromyalgia [164, 167, 182, 302]. The composition of gut microbiome on individuals varies greatly [303], emphasizing the importance of pre‐screening donor feces for an appropriate match between donor and recipient. Additionally, it should be noted that a single FMT treatment may not suffice as a cure, thereby necessitating multiple treatments for disease alleviation [304]. FMT is associated with relatively high costs for a singular treatment, which can result in elevated treatment expenses.

**Table 3:** The reported efficacies of probiotics in pain management.

Bacterial strains	Diseases	Functions	References
*Lactococcus lactis*	IBS	*L. lactis* carrying an expression plasmid for IL‐22 alleviated visceral hypersensitivity	[83]
*L. lactis*	IBS	*L. lactis* exerts antinociceptive properties through the production of GABA as an inhibitory neurotransmitter	[71]
*Lactobacillus reuteri*	IBS	Oral administration of *L. reuteri* improved visceral hypersensitivity by upregulating the expression of peripheral opioid receptors	[245]
*L. reuteri*	Neuropathic and inflammatory pain	No significant alleviation of neuropathic and inflammatory pain was observed after *L. reuteri* administration	[197]
*L. reuteri*	Infant colic	Feeding of *L. reuteri* promoted intestinal immune tolerance and mitigated infant colic	[292]
*L. reuteri*	GWI	Probiotic supplementation with *L. reuteri* functions as a regulator of exercise fatigue and cognitive impairment caused by GWI	[251]
*L. reuteri*	Capsaicin‐induced gut injury	Treatment with *L. reuteri* alleviated gut inflammation, restored the gut barrier, and increased the abundances of *Ruminococcaceae UCG_014* and *Akkermansia*	[293]
*L. reuteri*	Colitis	Administration of *L. reuteri* relieves colitis through histamine production and inhibition of IL‐6 and IL‐1β	[60]
*Lactobacillus paracasei*	IBS	Administration of *L. paracasei* attenuated visceral hypersensitivity induced by antibiotics	[100]
*L. paracasei* S16	Lumbar disc herniation with inflammatory pain	Administration of *L. paracasei* S16 regulates inflammation by decreasing pro‐inflammatory cytokines and purine metabolites	[177]
*L. paracasei* ET‐22	Dental caries	Administration of *L. paracasei* ET‐22 prevents dental caries by inhibiting the biofilm formation of microbiota	[223]
*Lactobacillus plantarum*	IBS	*Lactobacillus plantarum* therapy decreased serum corticosterone concentration and expression of mineralocorticoid receptors, thereby attenuating 5‐HT‐induced visceral hypersensitivity	[157]
*Lactobacillus acidophilus* DDS‐1	IBS	*L. acidophilus* DDS‐1 treatment significantly ameliorates abdominal pain	[294]
*L. acidophilus*	Osteoarthritis	*L. acidophilus* treatment regulated gut microbiota and mitigated joint pain	[175]
*Lactobacillus rhamnosus*	Widespread muscle pain	Administration of *L. rhamnosus* attenuated skeletal muscle pain	[295]
*L. rhamnosus*	Muscle mechanical hyperalgesia	*L. rhamnosus* elevated nociceptive thresholds in rats suffering from alcohol‐induced muscle mechanical hyperalgesia	[296]
*L. rhamnosus*	Bone cancer pain	*L. rhamnosus* attenuated mechanical allodynia and enhanced the analgesic effect of morphine through modulating gut microbiota in rats with bone cancer pain	[297]
*Bifidobacterium*	IBS	*Bifidobacterium* quadruple viable tablets ameliorated abdominal pain by increasing the abundance of SCFA‐producing bacteria and the concentration of SCFAs	[298]
*Bifidobacterium dentium*	IBS	*Bifidobacterium dentium* treatment modulates visceral sensitivity via GABA production	[70]
Heat‐inactivated *Bifidobacterium bifidum* MIMBb75	IBS	Heat‐inactivated *Bifidobacterium bifidum* MIMBb75 ameliorates serious abdominal pain adverse events	[159]
*Bifidobacterium longum* CECT 7347 and postbiotic heat‐treated *B. longum* CECT 7347	IBS	*B. longum* CECT 7347 and postbiotic heat‐treated *B. longum* CECT 7347 both alleviated visceral hypersensitivity	[158]
*Bifidobacterium infantis*	IBS	*Bifidobacterium infantis* significantly alleviated the pain sensation in patients with IBS that developed after a major flood disaster	[253]
*Bifidobacterium adolescentis* IPLA60004	Fibromyalgia	*Bifidobacterium adolescentis* IPLA60004 can convert glutamate to GABA, and reduce the symptoms associated with the excess of glutamate	[65]
Pasteurized *Akkermansia muciniphila*	IBS	Administration of pasteurized *Akkermansia muciniphila* alleviates visceral hypersensitivity by modulating intestinal permeability and microbiota homeostasis	[156]
*Roseburia hominis*	IBS	The administration of *Roseburia hominis* relieves visceral hypersensitivity through the production of butyrate	[155]
Golden bifid (*B. Longum*, *Lactobacillus bulgaricus* and *Streptococcus thermophilus*)	IBS	Treatment with Golden bifid alleviated patient symptoms and inhibited small intestinal bacterial overgrowth	[269]
Multiprobiotics	IBD	Probiotics alleviate clinical symptoms and may contribute to mucosal homeostasis in IBD dogs	[160]
Probiotics containing *L. paracasei*	Chronic constriction injury	Probiotics combined with adipose‐derived mesenchymal stem cells relieved neuropathologic pain	[196]
*Candida metapsilosis* M2006B	IBD	The addition of this fungal strain alleviated colitis by activating farnesoid X receptors	[286]
Multispecies probiotics containing 12 types	Migraine	Supplementation with multiple probiotics improves migraine characteristics, inflammatory markers, and intestinal permeability in female migraine patients	[203]
Multispecies probiotics (*Lactobacillus plantarum*, *Lactobacillus casei*, *L. acidophilus*, *Lactobacillus bulgaricus*, *Bifidobacterium infantis*, *B. longum*, *Bifidobacterium breve*, and *Streptococcus thermophilus*)	Migraine	The concurrent administration of probiotics and vitamin D alleviates migraine	[204]

Abbreviations: L. lactis, Lactococcus lactis; L. reuteri, Lactobacillus reuteri; L. paracasei, Lactobacillus paracasei; L. acidophilus, Lactobacillus acidophilus; L. rhamnosus, Lactobacillus rhamnosus; B. longum, Bifidobacterium longum.

Organoid and organs‐on‐a‐chip represent advanced *in vitro* systems. Since two‐dimensional cell lines cannot replicate human physiological processes, organoids and organs‐on‐a‐chip may help to reveal the complex interactions between microbiota and humans [305, 306]. Organoids can be cultured into 2D layers or 3D shapes [307]. Microbes live on the surface of the intestinal epithelium in 2D organoids, compared to 3D organoids where microbiota can propagate in the lumen. An innovative microfluidic device known as an organs‐on‐a‐chip offers precise regulation of physiological processes by segmenting microbiota through multiple scaffold surfaces [305, 307]. Noteworthy advancements have been made in pain research using organoid‐based studies to investigate the functional role of microbiota. Bellono et al. employed organoids to investigate the interaction between the intestinal epithelium and neurons. ECs secrete more than 90% 5‐HT of the body, yet identifying EC individually is challenging due to its minute size and low prevalence. By examining the receptors and signaling pathways of genetically marked ECs in intestinal organoids, associations between microbiota and pain have been elucidated [308]. Gao et al. conducted *in vitro* experiments by co‐culturing mast cells with colonic organoids. Their finding indicates that intestinal microbiota activate mast cells, resulting in increased mucosal 5‐HT level, which may be a potential cause of abdominal pain in patients with IBS [112]. In future investigations into pain treatments, utilizing organoid models holds great promise.

Multi‐omics technologies require a comprehensive application. Multi‐omics encompasses genomics, transcriptomics, proteomics, metabolomics, microbiomics, and so on. These methods have significantly enhanced our ability to analyze the intricate relationship between microbiota and pain phenomena [309]. Microbiomics specifically enables a thorough examination of the microbiome, providing insights into its interactions with both the environment and the host organism [310]. However, conventional measures such as α and β diversity reflect relative abundance indices that assess species diversity by evaluating community evenness and compositional shifts but fail to capture nuanced interactions between beneficial and pathogenic bacteria. The deployment of the F/B ratio represents a significant advancement by incorporating explicit microbial data for evaluating microbiota homeostasis. Technological advances have also facilitated the identification and analysis of microbial subpopulations. By merging pangenomics with metagenomics, we can gain insights not only into biogeographic patterns of individual populations but also into gene conservation across different populations [311]. Current advancements in microbial and pain research predominantly rely on integrating microbiomics with metabolomics while neglecting other omics approaches. Research has demonstrated that transplantation of the same microbe into different individuals through FMT leads to heritable changes in promoter orientations within a few days, demonstrating rapid adaptation in selective expression of microbial genes [312]. *Saccharomyces cerevisiae* actively alters tRNA abundance independent of mRNA levels to quickly adapt its metabolic state to environmental changes [313]. Transcriptomic and proteomic analyses help to understand rapid adaptation mechanisms employed by microbiota when faced with environmental changes [314, 315]. The advances in meta‐omics profiling provide more accesses to integrate biological information of prokaryotes [316]. Moreover, the integration of single‐cell sequencing and spatial omics technologies enhances the comprehensive characterization of individual cells and tissue architecture, thereby facilitating potential synergistic collaborations with microbiological investigations to drive innovative advancements in pain therapeutics [317, 318].

Integrate knowledge from diverse disciplines. For instance, even far away in the CNS, microglial maturation relies on the passage of SCFAs derived from intestinal microbiota through the vagus nerve [102], highlighting extensive functional connections between microbes, the immune system, and the nervous system. Metabolites originating from gut microbiota can influence intestinal neurons' function, immune cell activity, as well as interactions between immune and glial cells within the CNS. Several studies have demonstrated that microbial interventions reduce neuropathic inflammation and alleviate neuropathic pain by modulating the response of the immune system [200]. However, in the vast majority of current research, different disciplines are still fighting for themselves. “Two minds are better than one,” and the harmonious fusion of multiple disciplines emerges as a pivotal focus for future scholarly pursuits. For example, the efficacy of a variety of immunotherapy and biotherapy programs is related to the microbiota, and microbial intervention may be one of the ways to improve the efficacy [319, 320]. Another paradox is that the closer you look, the less you see. Although immune cells such as dendritic cells and natural killers play important roles in the intricate interplay with microorganisms and pain modulation, their precise functions have not yet been fully illuminated by current research. The utilization of novel technologies like omics approaches may propel advancements in related investigations.

## 7 CONCLUSIONS

Microbes play a pivotal role in pain modulation and have emerged as a prominent research topic. The dysbiosis of microbiota has been extensively documented in various diseases, encompassing both intra‐intestinal and extra‐intestinal conditions. Nociceptor neuron stimulation can be directly induced by microbially derived metabolites or indirectly triggered by microbially activated immune cells, as well as promptly initiated by microorganisms or their constituents. Deciphering the mechanisms through which microbes mediate pain opens up avenues for future investigations. Diagnostic panels based on microbiota detection consistently outperform conventional clinical methods in predicting and evaluating pain. Microbiological interventions targeting pain management have made significant strides towards clinical translation, exhibiting promising outcomes across diverse disease contexts such as dietary interventions, probiotics administration, and FMT. Nevertheless, existing limitations within current research impede further advancements in manipulating microbiota for pain modulation. This review proposes several crucial directions that hold promise for future investigations while emphasizing the integration of neurology, immunology, and microbiology to provide a comprehensive understanding of pain mechanisms. The intricate microbial ecosystem residing within the human body exerts profound influences on the perception of pain.

## AUTHOR CONTRIBUTIONS

**Huiguang Ren**: Writing—original draft; visualization; methodology; writing—review and editing. **Bo Cao**: Writing—original draft; writing—review and editing; methodology. **Qixuan Xu**: Methodology; Visualization; writing—review and editing. **Ruiyang Zhao**: Visualization; writing—review and editing. **Hanghang Li**: Writing—review and editing. **Bo Wei**: Writing—review and editing; conceptualization; supervision; funding acquisition.

## CONFLICT OF INTEREST STATEMENT

The authors declare no conflicts of interest.

## ETHICS STATEMENT

No animals or humans were involved in this study.

## Data Availability

No new data or scripts were generated in this review. Supplementary materials (graphical abstract, slides, videos, Chinese translated version and update materials) may be found in the online DOI or iMeta Science http://www.imeta.science/imetaomics/.
